# Prevention of atrial fibrillation after open-chest surgery with extracellular vesicle therapy

**DOI:** 10.1172/jci.insight.163297

**Published:** 2023-08-08

**Authors:** Sandrine Parent, Ramana Vaka, Yousef Risha, Clarissa Ngo, Pushpinder Kanda, Stanley Nattel, Saad Khan, David Courtman, Duncan J. Stewart, Darryl R. Davis

**Affiliations:** 1 University of Ottawa Heart Institute, Division of Cardiology, Department of Medicine, and; 2 Department of Cellular and Molecular Medicine, Faculty of Medicine, University of Ottawa, Ottawa, Ontario, Canada.; 3 Research Center and Department of Medicine, Montreal Heart Institute, University of Montreal, Montreal, Quebec, Canada.; 4 Department of Pharmacology and Therapeutics, McGill University, Montreal, Quebec, Canada.; 5 Institute of Pharmacology, West German Heart and Vascular Center, Faculty of Medicine, University of Duisburg-Essen, Essen, Germany.; 6 Ottawa Hospital Research Institute, Division of Regenerative Medicine, Department of Medicine, University of Ottawa, Ottawa, Ontario, Canada.

**Keywords:** Cardiology, Adult stem cells, Arrhythmias

## Abstract

Almost half of patients recovering from open-chest surgery experience atrial fibrillation (AF) that results principally from inflammation in the pericardial space surrounding the heart. Given that postoperative AF is associated with increased mortality, effective measures to prevent AF after open-chest surgery are highly desirable. In this study, we tested the concept that extracellular vesicles (EVs) isolated from human atrial explant-derived cells can prevent postoperative AF. Middle-aged female and male rats were randomized to undergo sham operation or induction of sterile pericarditis followed by trans-epicardial injection of human EVs or vehicle into the atrial tissue. Pericarditis increased the probability of inducing AF while EV treatment abrogated this effect in a sex-independent manner. EV treatment reduced infiltration of inflammatory cells and production of pro-inflammatory cytokines. Atrial fibrosis and hypertrophy seen after pericarditis were markedly attenuated by EV pretreatment, an effect attributable to suppression of fibroblast proliferation by EVs. Our study demonstrates that injection of EVs at the time of open-chest surgery shows prominent antiinflammatory effects and prevents AF due to sterile pericarditis. Translation of this finding to patients might provide an effective new strategy to prevent postoperative AF by reducing atrial inflammation and fibrosis.

## Graphical abstract

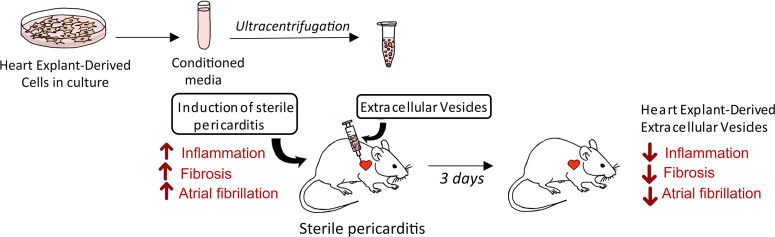

## Introduction

Atrial fibrillation (AF) is the most common heart rhythm disturbance in the world — afflicting almost 38 million patients worldwide (1–3). Although not usually acutely life-threatening, AF can significantly impact the quality of life for an otherwise-healthy patient by causing dizziness, fatigue, palpitations, and even syncope and is associated with increased mortality, heart failure risk, and stroke. For patients recovering from open-chest surgery on the heart or lungs, new-onset postoperative AF (POAF) can be difficult to manage, as many of the standard therapies (such as antiarrhythmics or anticoagulants) are contraindicated and routine postoperative medications (such as inotropes or vasopressors) increase the risk of AF, while complicating its management. It is thus unsurprising that new-onset POAF impacts surgical outcomes by increasing mortality, the length of inpatient stay, and overall procedural costs (4, 5). Given that almost half of all patients experience AF after cardiac surgery, regimes to prevent or treat POAF are highly desirable (6).

There is at present no effective solution to prevent POAF (7). Rhythm or rate control medications often fail and are limited by off-target effects on blood pressure or heart function (8, 9). Antiinflammatory medications increase the risk of hyperglycemia, infection, gastritis, and myelosuppression (10, 11). Antifibrotic approaches similarly impact postoperative healing and increase the risk of infection (12, 13). Given these considerations, recent preclinical work has focused on biological therapies to reduce inflammation or modify atrial electrophysiology. Despite promising results in animal models, no biological therapy has been translated to the clinic because of poor-quality evidence, modest efficacy, the impractical nature of the intervention, or the potential for complications.

Accordingly, we built on previous work from our group using human heart explant-derived cells (EDCs) (14–19) to establish the potential value of extracellular vesicles (EVs), which mediate the antifibrotic, antiinflammatory effects of transplanted cells, for the prevention of POAF. EDCs are CD105^+^CD45^–^ cardiac derived cells cultured from human atrial appendage biopsies obtained at the time of open-chest surgery. EDCs cultured in a Good Manufacturing Practice (GMP) cell manufacturing facility using serum-free, xenogen-free culture methods can be rapidly expanded to substantial doses (100 million or more cells in 2–3 weeks) that provide large numbers of EVs rich in miRNAs associated with attenuation of adverse cardiac fibrosis/remodeling and inflammation. In this report, we evaluated whether EVs collected from EDC producer cell lines can suppress atrial fibrosis, atrial inflammation, and AF promotion in a validated rodent model of postoperative sterile pericarditis (20, 21). We hypothesize that EVs would reduce atrial fibrosis and AF inducibility by reducing local inflammation and the malignant pro-fibrillatory transformation of atrial fibroblasts.

## Results

### Human atrial EVs contain antifibrotic/antiinflammatory transcripts and proteins.

**Figure 1:**
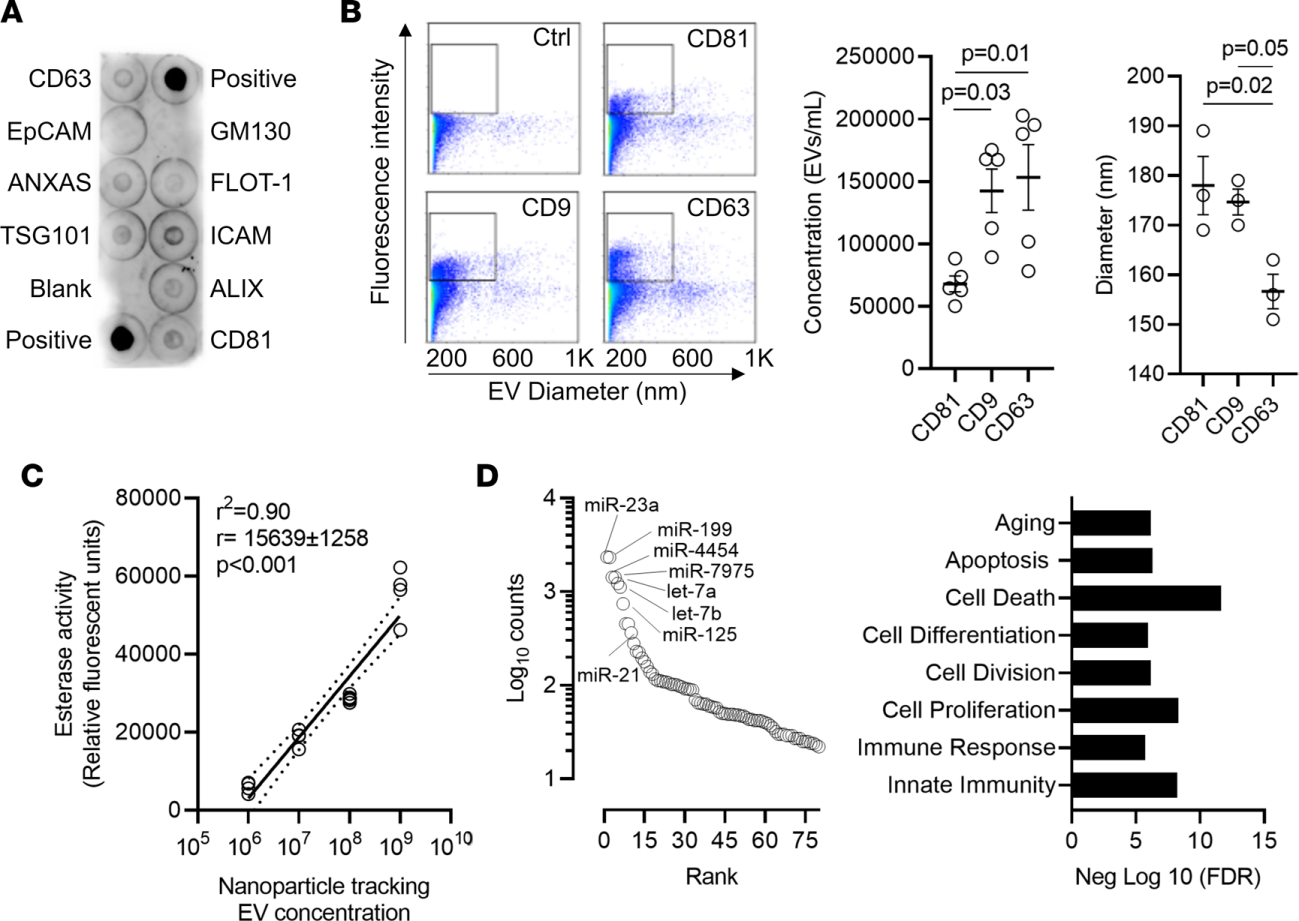
Characterization of extracellular vesicles produced by human atrial explant-derived cells cultured under serum-free, xenogen-free culture conditions within a clinical cell manufacturing facility. (**A**) Proteomic array demonstrating presence of markers indicative of EV identity while lacking cellular contaminants. (**B**) Flow cytometry demonstrating the relationship between EV size and surface marker expression (*n* = 3 biological replicates). One-way ANOVA with individual-mean comparisons by Bonferroni’s multiple 2-tailed comparisons test. (**C**) Simple linear regression of the relationship between EV concentration and acetylcholinesterase activity (*n* = 5 biological replicates). Data are plotted showing the 95% confidence bands of the best fit line. (**D**) Relative abundance of microRNA transcripts within EDC EVs (*n* = 3 biological replicates). EV, extracellular vesicles; FDR, false discovery rate; miR, microRNA.

Human EDCs were cultured in a clinical cell manufacturing facility from atrial appendage biopsies using serum-free, xenogen-free culture conditions. EVs were isolated from conditioned media after 48 hours in 1% oxygen, basal media conditions (21, 22). In keeping with accepted definitions, EDC EVs represented a polydisperse population of particles that ranged in diameter from 95 to 170 nm (mean ± SD 132 ± 7 nm). These microparticles contained transmembrane (CD63, CD81, FLOT1, ICAM1, EpCam) and cytosolic (ALIX, ANXA5, and TSG101) markers indicative of EV identity (23) while lacking evidence for cellular contaminants (GM130; Figure 1A). Flow cytometry demonstrated significant enrichment of microparticles with the prototypical EV markers CD9, CD63, and CD81 (Figure 1B and Supplemental Figure 1; supplemental material available online with this article; https://doi.org/10.1172/jci.insight.163297DS1). Interestingly, fewer particles expressed CD81 (~2-fold less, *P* < 0.05 vs. CD9 or CD63) while smaller particles were more apt to express CD63 (*P* < 0.05 vs. CD9 or CD81). Acetylcholinesterase activity was also present and correlated with nanoparticle particle tracking content (Figure 1C). These results verify the presence of a functional extracellular membrane–bound protein associated with EVs and provide a rapid means of confirming EV concentration.

**Figure 2:**
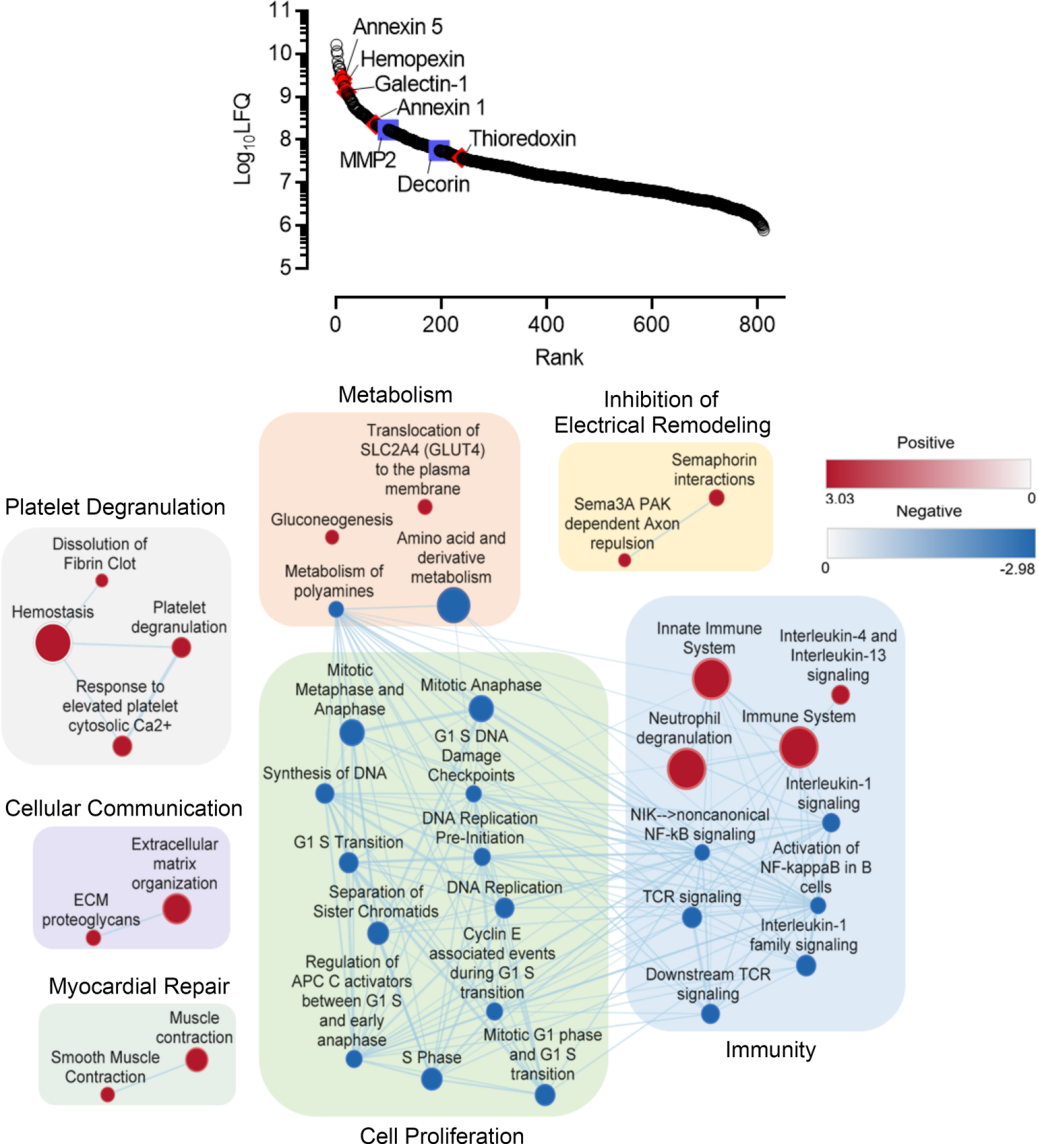
Human atrial explant-derived cells cultured under serum-free, xenogen-free culture conditions within a clinical cell manufacturing facility produce extracellular vesicles that contain antifibrotic/antiinflammatory proteins. Relative abundance of proteins within EDC EVs (*n* = 3 biological replicates). Enrichment map of the EDV EV proteome demonstrating the relationship between biological pathways that are expressed more than would be expected by chance. Pathways are represented by circles (nodes) connected with lines (edges) according to the number of shared proteins. Node size reflects the number of proteins participating in each pathway. The number of proteins shared by connected pathway nodes determines the thickness of edges. Upregulated pathways are colored red while downregulated pathways are in blue. The color of the concentric circles around each node represents the level of enrichment based on the enrichment score scale provided. Groupings of pathways are labeled by common activities or functions. APC, anaphase-promoting complex; ECM, extracellular matrix; LFQ, label-free quantification; MMP2, matrix metalloproteinase 2; NIK, NF-κB–inducing kinase.

The cargo within EDC EVs was enriched with 83 miRNA transcripts associated with reducing inflammation, stimulating angiogenesis, and suppressing fibrosis (Figure 1D and Supplemental Figure 2). Interestingly, the most abundant miRNAs (1,000+ counts) were associated with altered cell division/proliferation (let-7a, miR-23a, and miR-199a) and fibrosis (let-7a and miR-199a). Although EDC EVs contained miR-21, a transcript known to promote fibrosis and AF susceptibility (24), they lacked all other known pathological transcripts — miR-1 (25), miR-133 (26), miR-328 (27), and miR-590 (28) — and included miRNAs associated with reduced fibrotic atrial remodeling — miR-26 and miR-29 (29, 30). Within the 811 proteins identified within EDC EVs (Figure 2), the proteome was enriched with 196 proteins associated with reducing inflammation (Wilcoxon’s rank-sum test *P* < 0.003), which directly influenced both chemotaxis (i.e., annexin A1 and annexin 5; refs. 31–35) and macrophage function (i.e., galectin-1, hemopexin, and thioredoxin; refs. 36–40). Among the 28 proteins implicated in reducing fibrosis, decorin (a TGF-β1 inhibitor) and matrix metalloproteinase 2 were highly expressed (41–44). Enrichment mapping of the EV proteome predicted important roles in regulation of cell cycle, inflammation, and cellular signaling.

Taken together, these data support the notion that EDCs produce a defined EV product containing an antifibrotic, antiinflammatory cargo with the potential to alter the fundamental drivers of POAF.

### Intramyocardial injection of human EVs reduces inflammation, fibrosis, and fibrillation.

**Figure 3:**
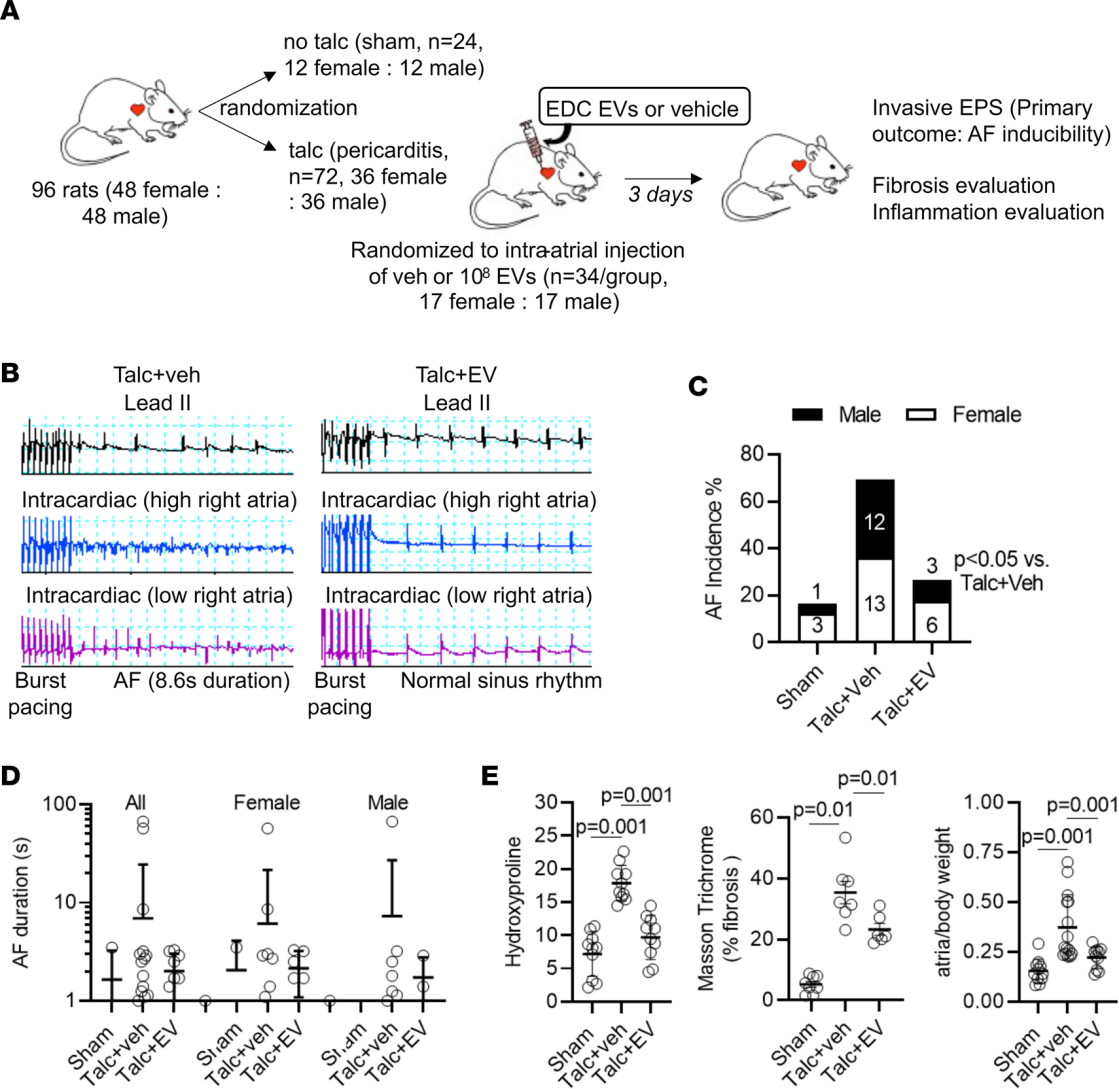
Human atrial extracellular vesicle effects on postoperative AF and atrial structure. (**A**) Study schematic demonstrating the study design, numbers of animals included, group allocations, and outcomes measured. One additional female rat was inadvertently randomized to receive extracellular vesicles (EVs) and was included in the final analysis (*n* = 18 female talc+EV group only). (**B**) Two representative tracings showing the effect of EV treatment on talc-treated animals after burst pacing. (**C**) Effect of EV treatment on the probability of inducing AF after burst pacing. Superimposed numbers indicate the absolute number of female (lower gray bar + number) or male (upper black bar + number) animals that went into AF. Logistic regression using a generalized linear model with binomial probability distribution and the logit link function was performed. (**D**) Effect of EV treatment on the mean duration of AF episodes (*n* = 2–25). (**E**) Effect of EV treatment on hydroxyproline content (*n* = 10), Masson’s trichrome fibrosis (*n* = 6–8), and weight (*n* = 10). One-way ANOVA with individual-mean comparisons by Bonferroni’s multiple 2-tailed comparisons test. AF, atrial fibrillation; EPS, electrophysiological study; EV, extracellular vesicles; veh, vehicle.

The antiarrhythmic potential of EVs against POAF was explored using a rat model of sterile pericarditis (20), whereby animals underwent open-chest surgery before randomization to epicardial application of talc or no talc (Figure 3A). Immediately after epicardial application of talc, animals were randomized again to trans-epicardial intramyocardial injection of EVs or vehicle (saline; Figure 3A) into the atrial tissue. As outlined in Supplemental Figure 3, all animals survived the initial surgery, while 3 animals died because of anesthetic overdose prior to the second procedure (all proving to be vehicle treated). Treatment with EVs reduced the probability of inducing AF 3 days after open-chest surgery by 40% (*P* < 0.01 vs. vehicle alone, Figure 3, B and C). Recipient sex did not alter this effect. In animals that experienced AF, neither EV treatment nor recipient sex significantly altered AF duration (Figure 3D), though the lack of statistical significance may be due to the very low number of sham- and EV-treated animals (4 and 9, respectively) that experienced AF and the nonparametric distribution of AF duration. If we allocate an AF duration of 0 to animals that did not experience AF (13), then EV treatment reduced the maximal AF duration, but interpretation of this result is inherently biased by the large numbers of animals that did not experience AF in the sham- and EV-treated groups (Supplemental Figure 4). Except for changes in P wave duration (a measure of atrial activation), pericarditis, EV treatment, and recipient sex had no effect on the electrocardiographic or electrophysiological measures of cardiac function (Table 1). As shown in Figure 3E, EV treatment attenuated atrial fibrosis (45% ± 19% reduction in hydroxyproline content, *P* = 0.01 vs. vehicle alone; 28% ± 19% reduction in Masson’s trichrome scar content, *P* = 0.01 vs. vehicle alone; Supplemental Figure 5; 30% ± 22% reduction in picrosirius red content, *P* = 0.01 vs. vehicle alone; Supplemental Figure 6) and enlargement (41% ± 15% reduction in atrial/body weight, *P* = 0.01 vs. vehicle alone), with the latter possibly reflecting increases in atrial fibrosis and cardiomyocyte hypertrophy (Supplemental Figure 7).

### EVs prevent inflammation and polarize atrial macrophages to a pro-healing phenotype.

**Figure 4:**
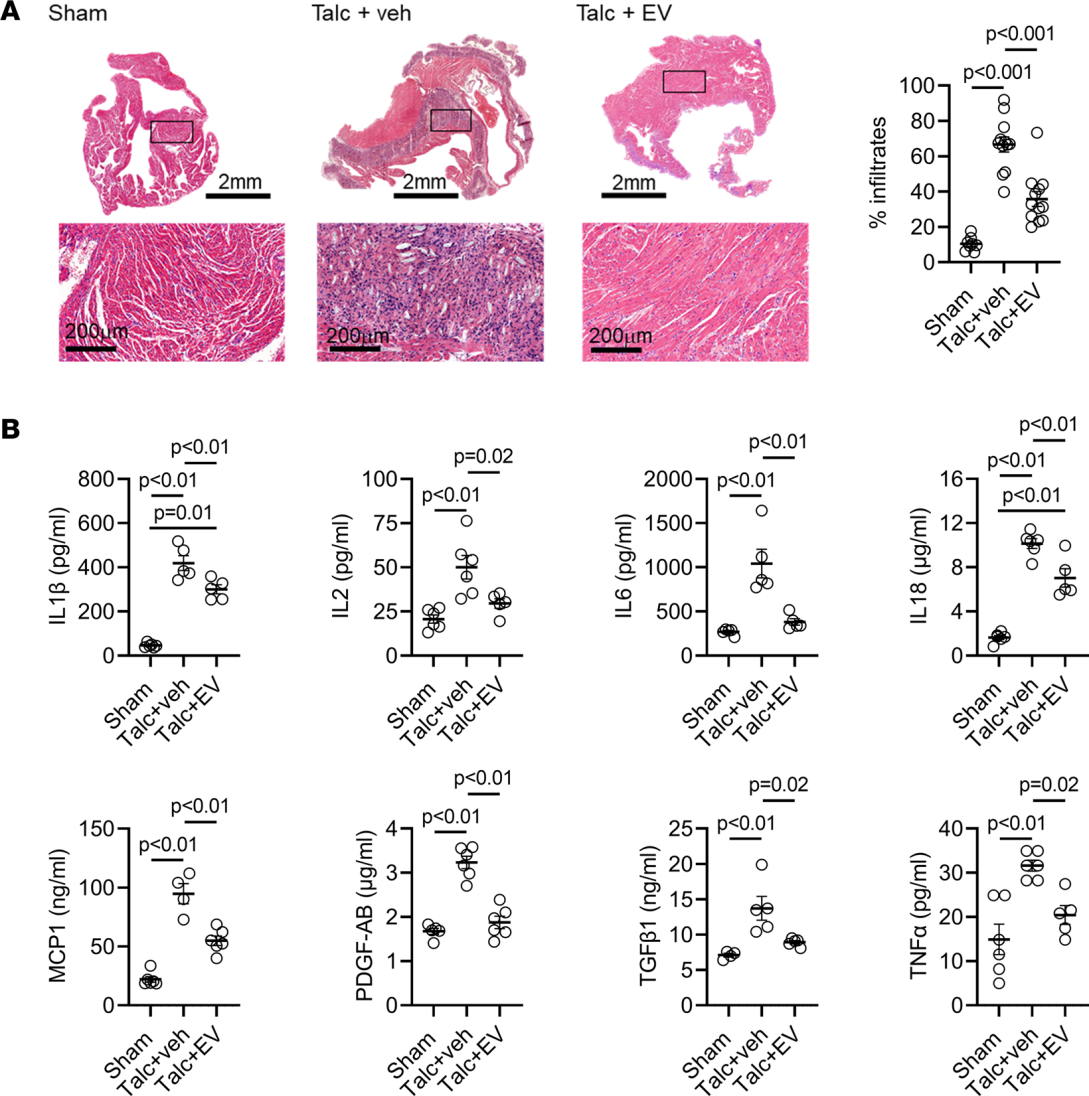
Effect of human atrial extracellular vesicles on inflammation. (**A**) Representative images of atrial tissue sections after hematoxylin and eosin staining showing the effect of talc and extracellular vesicles (EVs) on inflammatory cell infiltration. Quantitative analysis showing the effect of talc and EVs on inflammatory cell infiltration (*n* = 8–12 biological replicates). (**B**) Effect of talc and EVs on interleukin-1β (IL-1β), IL-2, IL-6, IL-18, monocyte chemoattractant protein–1 (MCP-1), platelet-derived growth factor AB (PDGF-AB), transforming growth factor-β1 (TGF-β1), and tumor necrosis factor-α (TNF-α) expression (*n* = 4–6 biological replicates). One-way ANOVA with individual-mean comparisons by Bonferroni’s multiple 2-tailed comparisons test. Scale bar, 2 mm or 200 μm, as indicated.

Open-chest surgery results in loss of the pericardial mesothelial cells, which provokes inflammatory infiltration and a fibrinous reaction (45). The influence of human EVs on inflammation was first evaluated using atrial histology of EV-treated rats. As shown in Figure 4A, sterile pericarditis markedly increased the inflammatory infiltrate detected in atrial sections (6-fold ± 2-fold increase, *P* < 0.001 vs. baseline). EV injection attenuated the inflammatory effect of talc as the inflammatory infiltrate was halved (47% ± 23% less; *P* < 0.001 vs. vehicle treatment). This decrease reflected the reduction in pro-inflammatory cytokines found in treated atria (Figure 4B). Sterile pericarditis alone resulted in prototypical increases of the pro-inflammatory cytokines IL-1β, IL-2, IL-6, IL-18, MCP-1, TGF-β1, PDGF-AB, and TNF-α. Intramyocardial injection of EVs prevented the rise in a number of these cytokines (IL-2, IL-6, PDGF-AB, TGF-β1, and TNF-α) while attenuating the observed increase in others (IL-1β, IL-18, MCP-1).

**Figure 5:**
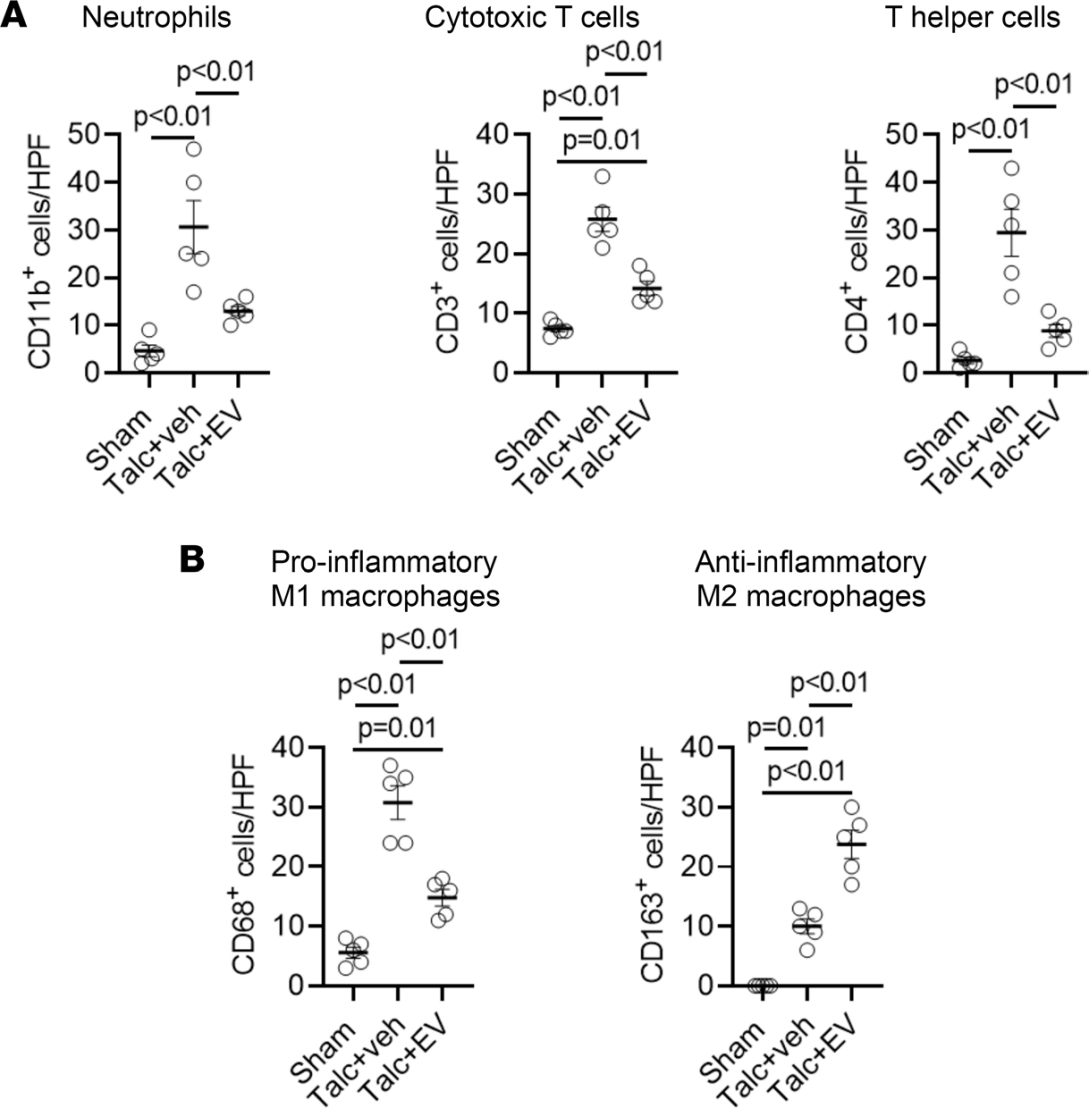
Effect of human atrial extracellular vesicles on inflammatory cell infiltration. (**A**) Effect of explant-derived cell (EDC) extracellular vesicles (EVs) on the number of neutrophils (CD11b^+^), cytotoxic T cells (CD3^+^), and T helper cells (CD4^+^) found within treated atria (*n* = 5 biological replicates, 6 random fields per biological replicate). (**B**) Effect of talc and EVs on the atrial content of pro-inflammatory M1 macrophages (CD68^+^) and antiinflammatory M2 macrophages (CD163^+^, *n* = 5 biological replicates, 6 random fields per biological replicate). One-way ANOVA with individual-mean comparisons by Bonferroni’s multiple 2-tailed comparisons test. HPF, high-power field.

To explore the mechanism underlying these observed changes, we profiled the inflammatory infiltrates found within the atria 3 days after surgery. As shown in Figure 5A and Supplemental Figures 8–10, sterile pericarditis increased the number of neutrophils (CD11b^+^), cytotoxic T cells (CD3^+^), and T helper cells (CD4^+^) in the atria. EV treatment significantly decreased the recruitment of all 3 cell types to near-baseline values. Sterile pericarditis also increased the atrial content of pro-inflammatory M1 macrophages expressing the surface marker CD68 by 4.5-fold ± 1.1-fold from sham (*P* < 0.01, Figure 5B and Supplemental Figure 11). Although M1 macrophages were also increased in EV-treated rats, this increase was significantly attenuated to 1.6-fold ± 0.5-fold (*P* = 0.01 vs. sham; Supplemental Figure 12). Interestingly, sterile pericarditis also increased the number of pro-healing antiinflammatory M2 macrophages (CD163-expressing) by 1.8-fold ± 0.5-fold from sham (*P* < 0.01). EV treatment markedly increased atrial content of CD163 macrophages (4.3-fold ± 1.0-fold greater, *P* < 0.01 vs. sham; *P* < 0.01 vs. vehicle-treated), suggesting that EV treatment promoted macrophage polarization toward an antiinflammatory phenotype that may help attenuate the inflammatory response.

The long-term consequences of sterile inflammation induced by talc were evaluated in a series of animals sacrificed 7 days after surgery (Supplemental Figure 13A). Pericarditis resulted in persistent chamber enlargement and increases in measures of fibrosis (hydroxyproline and Masson’s trichrome; Supplemental Figure 13, B and C). The changes mirrored persistent increases in inflammatory infiltrates (Supplemental Figure 14A) and inflammatory cytokines (Supplemental Figure 14B). Akin to the effects seen 3 days after surgery, EVs attenuated the effects of pericarditis on fibrosis, chamber enlargement, inflammatory infiltration, and inflammatory cytokine abundance.

### EVs directly prevent the activation of atrial fibroblasts.

**Figure 6:**
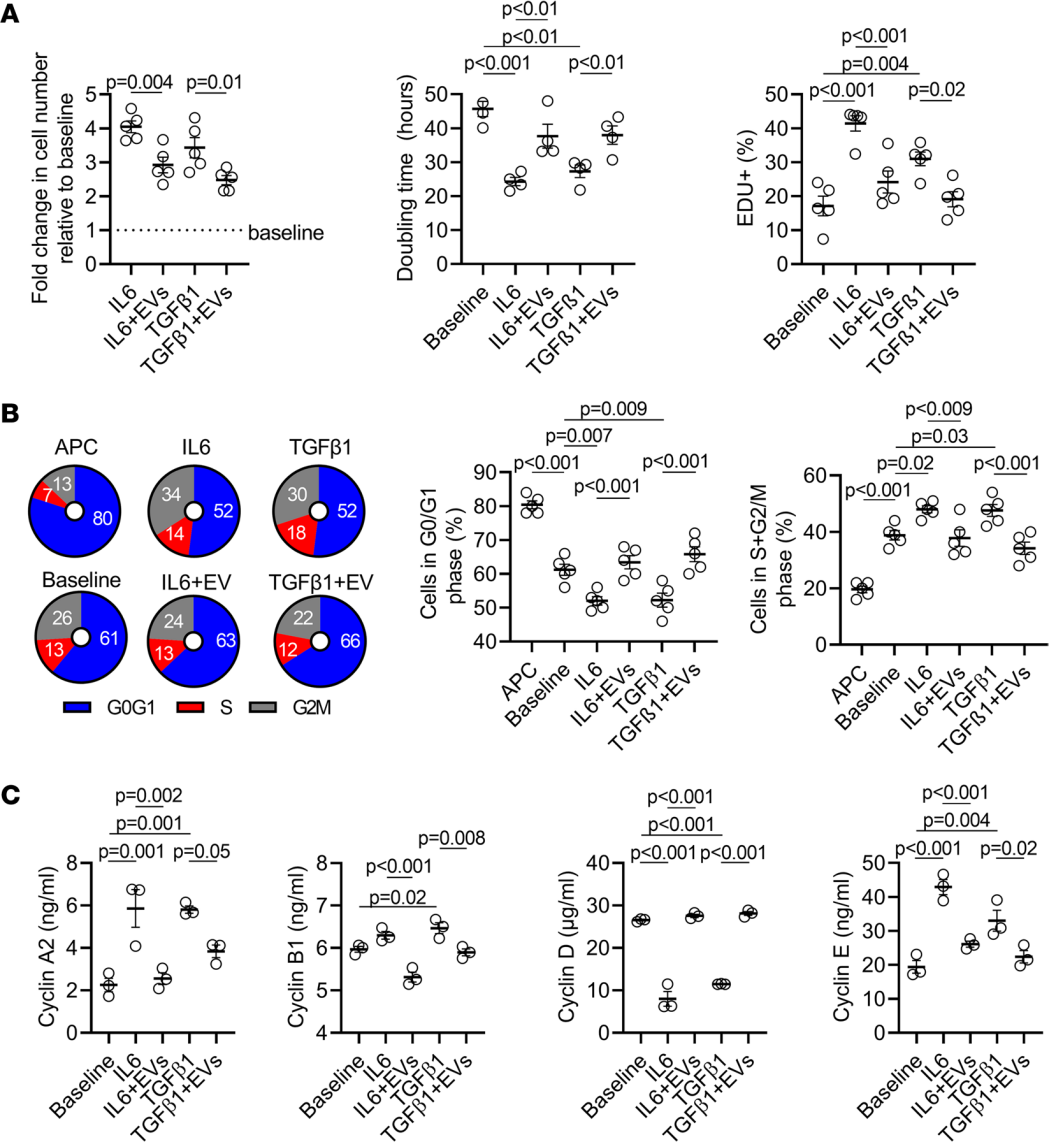
Effect of human atrial extracellular vesicles on atrial fibroblast proliferation. (**A**) Effect of explant-derived cell (EDC) extracellular vesicles (EVs) on the relative cell number, population doubling time, and nuclear incorporation of the thymidine analog 5-ethynyl-2′-deoxyuridine (EdU) within atrial fibroblasts at baseline and after treatment with interleukin-6 (IL-6), transforming growth factor β1 (TGF-β1), and/or EDC EVs (*n* = 5 biological replicates). Baseline indicates normal high serum conditions. (**B**) Cell cycle analysis showing the effect of anaphase-promoting complex (APC), IL-6, TGF-β1, and/or EDC EVs on the proportion of atrial fibroblasts in the G_0_/G_1_ or S and G2M phases of the cell cycle (*n* = 5 biological replicates). Baseline indicates normal high serum conditions. (**C**) Effect of IL-6, TGF-β1, and/or EDC EVs on proteomic expression of cyclins that control progression of atrial fibroblasts through the cell cycle (*n* = 3 biological replicates). Baseline indicates normal high serum conditions. One-way ANOVA with individual-mean comparisons by Bonferroni’s multiple 2-tailed comparisons test.

Fibroblasts comprise almost 75% of the cells within the heart (46). When fibroblasts are activated by profibrotic stimuli, they proliferate and differentiate into myofibroblasts, which reconfigure the extracellular matrix and can have adverse effects on atrial structure and electrophysiological function. Given that interfering with atrial fibroblast proliferation reduces fibrosis and AF burden, we explored the influence of EVs on atrial fibroblast proliferation. POAF was modeled by exposing normal rat atrial fibroblasts to IL-6 or TGF-β1 (47). As shown in Figure 6A and Supplemental Figure 15, IL-6 and TGF-β1 increased fibroblast proliferation as evidenced by increases in manual cell counts and EdU incorporation. Application of EVs at the time of IL-6/TGF-β1 exposure restored fibroblast proliferation to baseline.

Flow cytometry was used to profile the effects of EVs on cell cycle kinetics (Figure 6B). Fibroblasts grown in high serum conditions demonstrated progression through the cell cycle (G_0_/G_1_, S, and G2M phases). APC promoted degradation of cell cycle proteins, and cells accumulated in the G_0_/G_1_ phase. IL-6 and TGF-β1 reduced the proportion of fibroblasts within G_0_/G_1_ phase by 15% ± 4% (*P* = 0.004 vs. baseline) and 15% ± 8% (*P* = 0.005 vs. baseline) while increasing the proportion of cells in the proliferative S + G2M phases by 24% ± 7% (*P* = 0.01 vs. baseline) and 24% ± 12% (*P* = 0.01 vs. baseline), respectively. Coadministration of EVs abrogated the effects of each cytokine on the proportional changes in G_0_/G_1_ and S + G2M fibroblasts (*P* = NS vs. baseline). Fibroblast cultures were then interrogated to identify which cyclins within the regulatory machinery underlying cell cycle progression were modified by EV treatment. As shown in Figure 6C, treatment with IL-6 or TGF-β1 increased the content of cyclins A2, B1, and E while decreasing the content of cyclin D well below baseline values. Coadministration with EDC EVs attenuated these effects, with cyclin levels often normalizing after treatment. Interestingly, EV treatment decreased expression of cyclin B1 below baseline in a manner consistent with the observed effects on proliferation.

## Discussion

Conventional therapies for POAF reflect approaches to attain and maintain normal rhythm while preventing rate-related complications and systemic embolization (7). Despite these measures, a single occurrence of POAF often extends hospital length of stay and increases costs (4, 5). To date, only prophylactic beta blockers, which target the autonomic alterations associated with surgery, are routinely recommended to reduce the incidence of POAF (9). Given that the onset of POAF typically occurs 1 to 3 days after surgery, any therapy needs to be administered before the injury, influence function during this critical 1- to 3-day recovery period, and not have adverse effects on postoperative recovery. Here, we explored the concept that an intramyocardial dose of EVs injected into the atria would prevent inducible AF after open-chest surgery without a need for interventional procedures or systemic drugs. Atrial inflammation, one of the key drivers of POAF, was attenuated. In a manner consistent with the known antifibrotic effects of EVs on postinfarct ventricular remodeling (21, 22, 48–51), EVs rendered atrial fibroblasts inert to the pro-fibrotic effects of inflammation. The importance of these effects was manifested by a great reduction in AF inducibility 3 days after open-chest surgery.

The pathogenesis of POAF revolves about the interaction between preoperative, perioperative, and postoperative factors (52). Patients who are referred for surgery often present with a preexisting, adversely remodeled atrial substrate that reflects advanced age and other medical comorbidities. Surgery itself exposes patients to perioperative risks (such as anesthetics, atriotomy incisions, cardiopulmonary bypass, and electrolyte abnormalities) that further reduce the threshold for AF. Several days after surgery, autonomic nervous system activation, inflammation, oxidative stress, and atrial stretch combine to increase atrial vulnerability so that ectopic beats can initiate POAF. In this report, we test the idea that a single intramyocardial injection of EVs can attenuate postoperative factors that increase substrate vulnerability to AF, in particular inflammation and the pro-fibrillatory transformation of atrial fibroblasts. In support of this mechanistic link, we demonstrated that EVs reduced neutrophil infiltration, pro-inflammatory macrophage polarization, and inflammatory cytokine production. Conceptually, EVs must precondition resident atrial cells to limit inflammatory signaling. This mechanism is consistent with ventricular remodeling studies, which show that EVs from heart-derived cells change the polarization state of resident and infiltrating macrophages (16, 53), and with studies showing a role for atrial cardiomyocyte inflammatory signaling in POAF (54). Interestingly, a single dose of EVs also reduced atrial fibrosis, a component of the structural remodeling known to ensue after surgery (55). This in vivo effect may in part be attributable to inhibition of pro-inflammatory cytokines produced by resident cells, but in vitro profiling suggests that EV treatment renders atrial fibroblasts antifibrotic and resistant to pro-inflammatory stimuli (18). Thus, EV treatment leverages several mechanisms to reduce substrate vulnerability below the threshold at which triggers do not initiate POAF.

These results contrast with other preclinical approaches to POAF in several ways. First, our study design fulfills many of the SYRCLE criteria used to exclude bias (56) and many of the CAMARADES checkpoints used to evaluate study quality (57). We also chose to examine the clinically meaningful “incidence of inducible AF” for our primary outcome rather than total duration of AF. Unlike most preclinical studies to date (12, 13), the scalable biological product used in this study was cultured to GMP cell manufacturing standards using sourced xenogen-free materials, which makes clinical translation relatively straightforward. To maximize benefit to the treated substrate, we evaluated a biological product that simultaneously targets many mechanistic pathways known to contribute to POAF vulnerability rather than focusing on a single mechanism that may leave a host of other fundamental changes initiating and maintaining AF unaddressed (29, 58–63). In contrast to a drug or a single miRNA transcript, EVs are manufactured by producer cell lines. As such, they provide a platform amenable to engineering refinements. Although EDC EVs possess many miRNA transcripts known to reduce fibrotic atrial remodeling (miR-26 and miR-29; refs. 29, 30), they also contain the pro-arrhythmic transcript miR-21 (24). Thus, relatively simple engineering of producer cell lines to knock down pathological transcripts within EVs has the potential to further enhance the antiarrhythmic potency, a concept requiring further experimental assessment. Finally, systemic therapies may have unwanted off-target effects, such as impaired wound healing, infection, hyperglycemia, gastritis, pro-arrhythmia, or myelosuppression. The demonstration that a single injection of EVs directly into the target tissue provides marked, lasting benefits is interesting because such a local approach is unlikely to be limited by off-target adverse effects in remote tissues.

Despite these promising features and results, our study has several potential limitations that need to be acknowledged. Although rodents provide a cost-effective means of testing new therapies, their repolarizing ion channel profile and basal heart rates differ from those of humans. Rodent hearts are also inherently much smaller and do not generally exhibit spontaneous AF, requiring AF induction to obtain indices of the AF substrate. Our findings rationalize future work using large animal models that better reflect human electrophysiology and provide the opportunity for realistic clinical scaling. We also recognize that the extent to which EVs may act upon the preoperative components of the vulnerable substrate for AF is speculative and not the focus of this study, as none of the treated animals had a preexisting atrial cardiomyopathy. However, this approach has merit, as similar studies looking at delayed administration of EVs after myocardial ischemia (17, 48) suggest that treatment has the potential to promote salutary atrial remodeling, which may reduce vulnerability to AF. Similarly, the extent to which EV treatment alters the substrate effects of cardiac incision, open-heart surgery, and cardiopulmonary bypass–related systemic inflammation was not examined in this model, as none of the animals had surgical cardiac lesions or underwent cardiopulmonary bypass. Our analysis of atrial hypertrophy focused on 2 key measures, namely increased fibrosis and the cross-sectional area of individual atrial myocytes. However, we acknowledge a limitation in accurately quantifying the latter due to the inherent challenge of controlling for fiber orientation. Finally, analysis supporting the extent to which EVs alter inflammatory infiltrates was limited to histological section alone as the small atrial mass precluded more quantitative analysis, such as flow cytometry. Follow-up studies in complimentary large animal models are needed to confirm these findings (64).

In summary, our study indicates that a single dose of EVs delivered at the time of open-chest surgery directly into the affected area (i.e., the atria) before the disease starts has the potential to target multiple pathways resulting in AF. Our published work has shown that scaling using serum-free, xenogen-free techniques is feasible and will provide a cost-effective product for clinical use. This approach merits future attention as assessment in patients would be relatively straightforward, the clinical problem addressed is quite significant, and clinical event rates are high.

## Methods

### In vivo study design.

To reflect the real-world context and probe for sex-based (biological) differences, the study was performed in both female and male middle-aged rats. Middle-aged Sprague-Dawley rats (6 months old, Charles River Laboratories) of both sexes underwent induction of sterile pericarditis or sham operation under a protocol approved by the University of Ottawa Animal Care Committee. The detailed protocol was registered a priori within the Open Science Framework (https://osf.io/v9j63/?view_only=0e0ce767cce74bdb8c4fac1b0769a447). A 1-way study was designed to test if intramyocardial injection of EDC EVs at the time of sterile pericarditis induction significantly reduced the incidence of AF at invasive electrophysiological testing (primary outcome). A rat model of sterile pericarditis following talc application was used (20). To increase the rigor and reproducibility of the study, we studied 6-month-old female and male rats. We assumed that the incidence of AF would be 0.6 after talc treatment and that EV treatment would reduce AF incidence to 0.2. Sex was assumed not to alter the incidence of inducible AF. Based on these assumptions, group sample sizes of 34 rats (17 female + 17 male) would achieve an 83% power to detect superiority over vehicle using a 2-sided Mann-Whitney test (probability of a false positive result [α error] = 0.05).

### EDC culture and EV isolation.

Human EDCs were obtained from left atrial appendages donated by patients undergoing clinically indicated heart surgery and giving informed consent under a protocol approved by the University of Ottawa Heart Institute Research Ethics Board. EDCs were cultured using serum-free, xenogen-free methods in the Ottawa Hospital Clinical Cell Manufacturing Facility as previously described (21, 49, 50). Briefly, cardiac biopsies were minced, digested (Roche), and plated within Nutristem media (Biological Industries) exposed to physiologic (5%) oxygen in a GMP cell manufacturing facility. Once a week for 4 weeks, EDCs were collected from the plated tissue using TrypLE Select (Thermo Fisher Scientific) for direct experimentation.

Conditioned medium was collected after 48 hours of culture in 1% EV-depleted serum (System Biosciences) and 1% oxygen for centrifugation at 10,000*g* for 30 minutes and 100,000*g* for 3 hours at 4°C to pellet EVs (22, 51). EV content, size, and surface marker expression were analyzed using acetylcholinesterase activity (Fluoro-Cet, Systems Biosciences), nanoparticle tracking (Nanosight), and candidate antibody array (Exo-Check, Systems Biosciences) analysis.

### EV miRNA and proteome analysis.

The miRNA content within EDC EVs was profiled using multiplex fluorescent oligonucleotide-based miRNA detection (Human v3, NanoString), as previously described (22, 51). Briefly, miRNA was extracted (miRNeasy, QIAGEN) and quantified (2100 Bioanalyzer, Agilent) prior to profiling (Counter Human V3 miRNA Expression Assay, NanoString). Quality of images was evaluated (nSolver), which were discarded if the percentage field of view and sample binding density exceeded prespecified standards. Background subtraction was performed using the mean of negative controls plus 2 SDs. Counts were normalized using trimmed mean of M values, and differentially expressed miRNAs were identified using the generalized linear model likelihood ratio test.

EDC EVs were lysed [8 M urea, 100 mM 4-(2-hydroxyethyl)-1-piperazineethanesulfonic acid, 5% glycerol, and 0.5% n-dodecyl-β-d-maltoside; Thermo Fisher Scientific], reduced [tris(2-carboxyethyl) phosphine alkylated with iodoacetamide], and digested (trypsin/Lys-C solution, Promega) prior to formic acid treatment, desalination (C18 TopTips, Glygen), and vacuum drying. Protein samples were analyzed using an Orbitrap Fusion mass spectrometer (Thermo Fisher Scientific) coupled to an UltiMate 3000 nanoRSLC (Dionex, Thermo Fisher Scientific) as previously described (65). Using MaxQuant software, peptides were searched against the human UniProt FASTA database with a false discovery rate of 1%. Pathway analysis terms were extracted from the Reactome database (66) for network analysis (Cytoscape). Only proteins found in at least 2 biological replicates were considered for analysis.

Flow cytometry was performed on EVs that were individually labeled for CD9 (312106, BioLegend), CD63 (353004, BioLegend), or CD81 (349506, BioLegend) using a CytoFLEX S Beckman Coulter flow cytometer (67). Light scatter was calibrated using the National Institute of Standards and Technology Traceable Size Standards (Thermo Fisher Scientific) while fluorescence was calibrated using Molecules of Equivalent Soluble Fluorochrome beads (BD Biosciences) for analysis using FCMPASS (v3.07, NIH National Cancer Institute) and FlowJo (V10.7, BD Biosciences) (68). The details of EDC EV characterization have been submitted to the EV-TRACK knowledgebase (EV-TRACK ID: EV210347) (68, 69).

### Atrial fibroblast isolation.

Primary cultures of rat atrial fibroblasts were isolated from the hearts of middle-aged Sprague-Dawley rats (6 months old, Charles River Laboratories) using enzymatic digestion (Collagenase Type II, Worthington Biochemical) at 37°C. Cells were cultured in Dulbecco’s Modified Eagle High Glucose Medium (Thermo Fisher Scientific), supplemented with 10% fetal bovine serum (Thermo Fisher Scientific), 1% l-glutamine (Thermo Fisher Scientific), and 1% penicillin-streptomycin (Thermo Fisher Scientific). Second or third passage fibroblasts were used in all subsequent experiments.

### Surgical procedures.

Rats were fed rat chow and housed under a 12-hour light/12-hour dark cycle at 21°C and 50% humidity. All animals had free access to tap water and food. After preoperative buprenorphine (0.03 mg/kg subcutaneous), rats were anesthetized with 3% isoflurane, intubated, and ventilated. The thorax was shaved and sterilized with 2% w/v chlorhexidine gluconate in 70% v/v isopropyl alcohol. Animals were then randomized to sham operation (*n* = 24; 12 female, 12 male), induction of sterile pericarditis with intramyocardial injection of 10^8^ atrial EVs (*n* = 35; 18 female, 17 male), or induction of sterile pericarditis with intramyocardial injection of vehicle (*n* = 34; 17 female, 17 male) using a sealed envelope approach. Animals randomized to sterile pericarditis underwent a thoracotomy, and the atrial surfaces were dusted with sterile talcum powder (Thermo Fisher Scientific). Animals randomized to a sham procedure underwent a superficial incision that was closed in a manner indistinguishable from thoracotomy animals. Intramyocardial injections were performed using a total volume of 100 μL injected using a Hamilton microsyringe (27-gauge needle) into the left atrial wall at 5 separate injection points (24). Injections into the atria were done as superficially as possible. During the injections, a bleb was visible under the epicardial surface, reminiscent of the small blister that forms under the skin when performing an intradermal injection. The needle was then retained at the site of injection for 5 seconds to prevent leakage from the site of injection. Prior to closing the chest, the edges of the pericardium were closely approximated but not sutured together. After surgery, animals were placed in a 30°C incubator with supplemental oxygen and moistened food until they returned to a physiological state. Additional doses of buprenorphine (0.03 mg/kg subcutaneous) were administered 6 and 12 hours postoperatively. A University of Ottawa Animal Care Technician monitored animals twice daily for 2 days after surgery. Investigative staff were masked to the treatment received, and analysis was conducted by individuals masked to group allocation. Group allocations were kept in a separate password-protected list for unmasking after analysis of the primary study outcome was completed. Ninety-six rats underwent surgery, and all completed the study with no adverse events or protocol deviations (Supplemental Figure 3).

Three days after surgery, all rats underwent invasive electrophysiological testing (24). After intraperitoneal injection of sodium pentobarbital (40 mg/kg), a 1.6F octopolar catheter (Millar) was inserted into the right atrium via the jugular vein for stimulation and recording. The surface electrocardiogram (lead I and II) and intracardiac electrograms (octopolar catheter 1/2, 3/4, 5/6, and 7/8) were continuously simultaneously monitored (ADInstruments). The atrioventricular nodal refractory period was determined as the longest S1–S2 interval that failed to conduct to the ventricle using twice-threshold, 2 ms, square-wave pulses after a 10-stimulus drive train (S1, 100 ms cycle length) followed by an S2 decremented in 2 ms intervals. If that failed to induce AF, 10–30 seconds of atrial burst pacing was performed at cycle lengths that ranged between 20 and 80 ms. AF was defined by rapid and fragmented atrial electrograms without discernible P waves on the surface electrocardiogram and with an irregular ventricular rhythm that lasted for at least 500 ms (70). AF duration was defined as longest single episode recorded. These data are displayed as the average of the longest episodes of AF for each group of animals. To probe for an effect of EV treatment on the duration of AF, animals that did not experience AF were not included in this average. At the end of the study, rats were sacrificed by exsanguination under pentobarbital anesthesia after displaying absence of withdrawal reflex to toe pinch.

A separate cohort of female rats underwent surgery to evaluate the persistence of talc ± EV effects on fibrosis and inflammation (*n* = 15 per group). Seven days after surgery, animals were sacrificed under pentobarbital anesthesia after displaying absence of withdrawal reflex to toe pinch.

### Histological analysis and quantification of fibrosis.

Atria were fixed and sectioned for histological analysis of inflammatory infiltrates by staining for hematoxylin and eosin (MilliporeSigma), activated T lymphocytes (CD3, ab16669, Abcam; CD4, ab237722, Abcam), macrophage infiltration/polarization (CD68, ab125212, Abcam; CD163, ab182422, Abcam), and neutrophil infiltration (CD11b, ab133357, Abcam). Hematoxylin and eosin staining was quantified using the ImageJ color deconvolution plugin (48, 71). Wheat germ agglutinin (Thermo Fisher Scientific), which selectively binds the cell membrane of mammalian cells, was used to identify cell borders. Left atrial collagen was quantified based on hydroxyproline content measurement (K555-100, BioVision) (72, 73). Atrial fibrosis was verified using Masson’s trichrome (Thermo Fisher Scientific) and picrosirius red staining (Thermo Fisher Scientific) (21, 49, 50).

### Inflammatory cytokines.

Atrial tissue was minced and homogenized using a tissue homogenizer (TissueRuptor, QIAGEN) according to the manufacturer protocol. Inflammatory cytokines and chemokines were measured using a multiplex Luminex-based assay (LXSARM, R&D Systems). Each sample was run in duplicate in a 96-well plate. IL-1β, IL-2, IL-18, and TNF-α were measured using a MAGPIX system (C4447b, Luminex). Acquired mean fluorescence data were analyzed and calculated by xPONENT software. IL-6 (ERA32RB, Invitrogen), TGF-β1 (ab119558, Abcam), PDGF-AB (ab213906, Abcam), and MCP-1 (ab100778, Abcam) were quantified using commercially available ELISAs according to the manufacturers’ protocols.

### Fibroblast proliferation.

The POAF cellular context was modeled by exposing normal rat atrial fibroblasts to IL-6 or TGF-β1 to recapitulate the inflammatory in vivo environment (47). Proliferation of atrial fibroblasts at baseline and after treatment with IL-6, TGF-β1, and/or EDC EVs was evaluated by staining for the nuclear incorporation of the thymidine analog EdU (17-10525, MilliporeSigma) and DAPI (MilliporeSigma). Manual cell counts were performed to verify the findings. Population doubling time was measured with a colorimetric assay (Dojindo). Cell cycle distribution was evaluated via flow cytometry according to the manufacturer’s instructions (4500-0220, Guava, MilliporeSigma). Briefly, fibroblasts were cultured for 24 hours with 10^8^ atrial EVs, IL-6, TGF-β1, a G1 cell arrest control (aphidicolin, MilliporeSigma), or vehicle. Flow cytometry was performed to quantify populations within G_0_/G_1_, S, and G2M phases of the cell cycle for each treatment (Guava, MilliporeSigma). To further explore the effect of EDC EVs on atrial fibroblasts, commercially available ELISAs were performed to look at the molecular regulators of cell cycle progression. Atrial fibroblasts were lysed according to the manufacturer’s instructions, and ELISAs were performed for cyclin A2 (MBS7211946, MyBioSource), B1 (MBS9328611, MyBioSource), D (MBS721009, MyBioSource), and E (MBS1600300, MyBioSource).

### Statistics.

All statistical tests used and graphical depictions of data (means and error bars or box-and-whisker plots) are defined within the figure legends for the respective data panels. All data are presented as mean ± SEM. To determine if differences existed between groups, data were first analyzed by 1-way ANOVA (SPSS v20.0.0); if among-group differences existed, Bonferroni’s corrected 2-tailed *t* test was used to determine the statistical significance of intergroup differences. In all cases, variances were assumed to be equal, and normality was confirmed prior to post hoc testing. Differences in categorical measures were analyzed using a χ^2^ test. A final value of *P* ≤ 0.05 was considered significant for all analyses.

### Study approval.

Human atrial appendage samples were obtained from patients undergoing cardiac surgery. Written informed consent was obtained from all participants. The study protocol was approved by the University of Ottawa Heart Institute Research Ethics Board (Protocol ID 20150313-01H), and the study was conducted in accordance with the principles of the Declaration of Helsinki. All animal study procedures and experiments were reviewed and approved by the University of Ottawa Animal Care Committee (HI-3137).

### Data availability.

Values for all data points found in graphs are in the Supporting Data Values file.

## Author contributions

DRD, SN, DJS, SP, SK, and DC designed the experiments. DRD, SP, DJS, and SN wrote the research plan and the animal protocols for the studies. DC and SK performed EDC culture in the cell manufacturing facility. RV analyzed the miRNA data. YR performed the proteomic studies and analyzed the data. SP, CN, and PK were responsible for the generation and analysis of in vitro study data. SP and DRD performed the animal studies. SP, CN, PK, and DRD analyzed the data. SP, RV, YR, and DRD cowrote the manuscript. All authors reviewed and approved the final version of the manuscript.

## Supplementary Material

Supplemental data

Supporting data values

## Figures and Tables

**Table 1:**
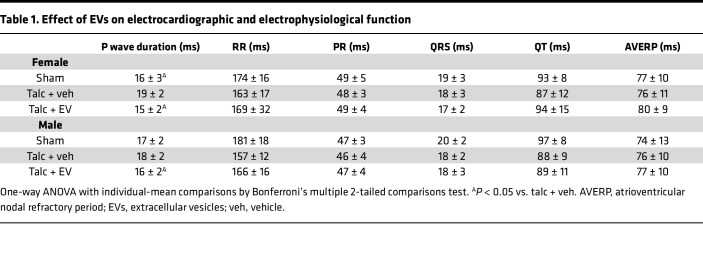
Effect of EVs on electrocardiographic and electrophysiological function

## Version 1 06/29/2023

In-Press Preview

## Version 2 08/08/2023

Electronic publication
