# Supplementary material for: Prevention of atrial fibrillation after open-chest surgery with extracellular vesicle therapy
Source: JCI Insight. 2023 Aug 8;8(15):e163297. doi: 10.1172/jci.insight.163297 (PMC10481795; doi:10.1172/jci.insight.163297)
Supplement: Supplemental data [file jciinsight-8-163297-s008.pdf]

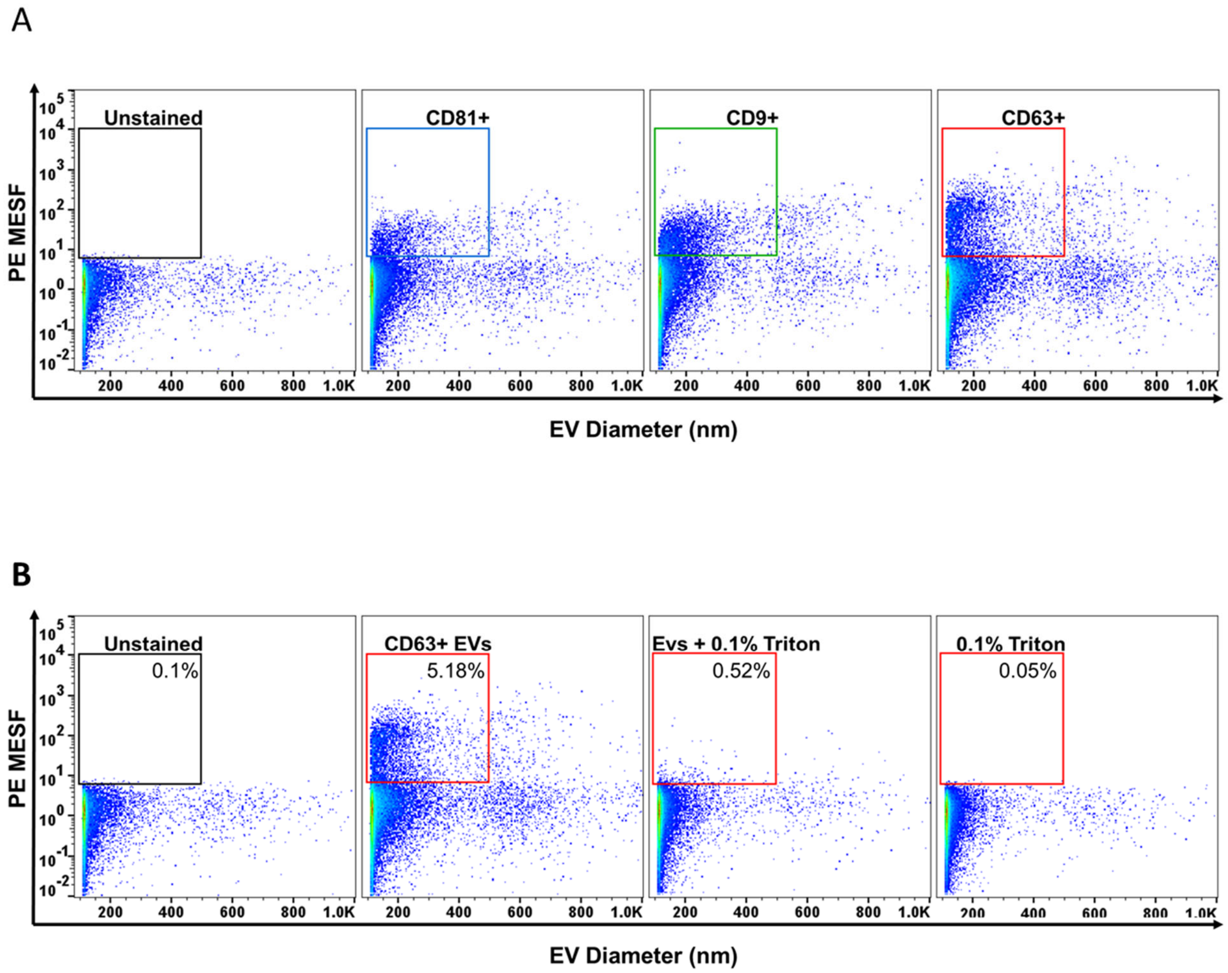

Supplemental Figure 1. Flow cytometry of extracellular vesicles. (**A**) Representative images showing the relationship between extracellular vesicle (EV) diameter and surface marker expression. EVs within a range of 100 – 500 nm in diameter are gated. Fluorescence intensity is quantified using standard units known as Molecules of Equivalent Soluble Fluorochrome (MESF). (**B**) Representative image of flow cytometry demonstrating EV depletion after solubilization. The addition of 0.1% Triton X-100 solubilizes CD63 stained EVs. Number inside the gates represent percentage of gated population within all particles detected. PE MSEF, Phycoerythrin Molecules of Equivalent Soluble Fluorochrome.

	Average counts
hsa-miR-23a-3p	2355
hsa-miR-199a-3p+hsa-miR-199b-3p	2326
hsa-miR-4454+hsa-miR-7975	1426
hsa-let-7a-5p	1221
hsa-let-7b-5p	1122
hsa-miR-125b-5p	743
hsa-miR-100-5p	453
hsa-miR-29b-3p	450
hsa-miR-21-5p	362
hsa-miR-191-5p	279
hsa-miR-199b-5p	230
hsa-miR-29a-3p	224
hsa-miR-22-3p	195
hsa-let-7i-5p	178
hsa-miR-181a-5p	159
hsa-miR-25-3p	139
hsa-miR-127-3p	131
hsa-let-7g-5p	117
hsa-miR-15b-5p	112
hsa-miR-320e	112
hsa-miR-221-3p	109
hsa-let-7d-5p	108
hsa-miR-16-5p	106
hsa-miR-424-5p	102
hsa-miR-3180	102
hsa-miR-374a-5p	101
hsa-miR-15a-5p	98
hsa-miR-130a-3p	96
hsa-miR-376a-3p	92
hsa-miR-199a-5p	92
hsa-miR-222-3p	90
hsa-miR-4286	89
hsa-miR-4516	70
hsa-miR-34a-5p	65
hsa-miR-1255a	64
hsa-miR-365a-3p+hsa-miR-365b-3p	63
hsa-miR-323a-3p	60
hsa-let-7c-5p	59

	Average counts
hsa-miR-27b-3p	58
hsa-miR-134-3p	57
hsa-miR-451a	52
hsa-miR-23b-3p	50
hsa-miR-423-5p	50
hsa-miR-28-5p	49
hsa-miR-125a-5p	49
hsa-miR-24-3p	49
hsa-miR-382-5p	48
hsa-miR-1228-3p	48
hsa-miR-20a-5p+hsa-miR-20b-5p	47
hsa-let-7e-5p	45
hsa-miR-18a-5p	43
hsa-miR-337-5p	43
hsa-miR-320e	42
hsa-miR-106a-5p+hsa-miR-17-5p	42
hsa-miR-19b-3p	41
hsa-miR-140-5p	40
hsa-let-7f-5p	39
hsa-miR-323a-5p	37
hsa-miR-148a-3p	35
hsa-miR-132-3p	32
hsa-miR-136-5p	30
hsa-miR-376c-3p	30
hsa-miR-379-5p	30
hsa-miR-26a-5p	29
hsa-miR-202-3p	29
hsa-miR-1290	29
hsa-miR-154-5p	27
hsa-miR-214-3p	27
hsa-miR-377-3p	27
hsa-miR-381-3p	25
hsa-miR-188-5p	25
hsa-miR-26b-5p	25
hsa-miR-363-3p	24
hsa-miR-337-3p	24
hsa-miR-137	23

Supplemental Figure 2. MicroRNA transcript expression within explant-derived cell extracellular vesicles.

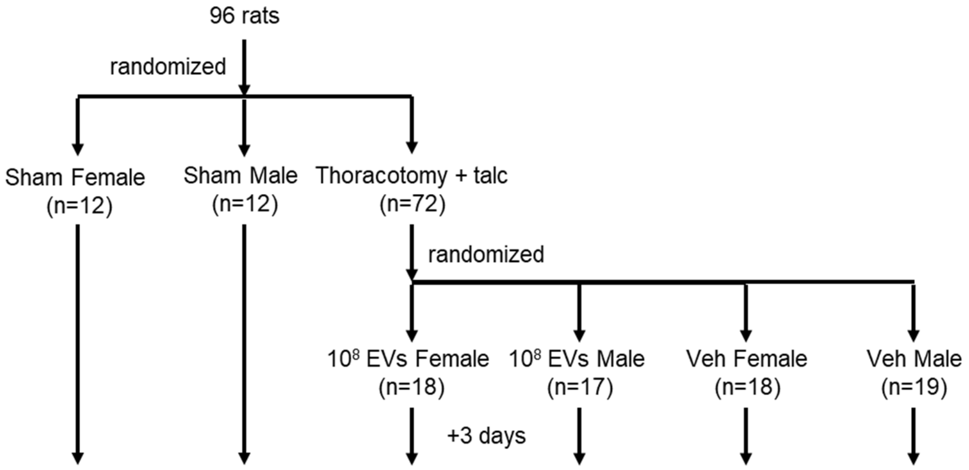

	Sham Female	Sham Male	10^8 EVs Female	10^8 EVs Male	Veh Female	Veh Male
**Adverse outcomes**						
Died after initial surgery	0	0	0	0	0	0
Died after second surgery	0	0	0	0	1	2
**Primary outcome**						
In vivo EPS:	12	12	18	17	17	17
**Secondary outcomes**						
Histological analysis:	8		12		12	
Hydroxyproline analysis:	10		10		10	
Inflammatory cytokines:	6		6		6	

Supplemental Figure 3. In vivo study conduct and group sizes. We assumed the incidence of atrial fibrillation (AF) would be 0.6 after talc treatment and that extracellular vesicle (EV) treatment would reduce AF incidence to 0.2. Sex was assumed not to alter the incidence of inducible AF. Based on these assumptions, group sample sizes of 34 rats (17 female + 17 male) would achieve an 83% power to detect superiority using a two-sided Mann-Whitney test (probability of a false positive result (alpha error) = 0.05). Animals that died because of anesthetic overdose prior to the second procedure were not included in the analysis. An allocation error resulted in 1 additional female rat receiving EVs. To avoid compromising the data, the animal was included in the analysis. Given that recipient sex did not influence occurrence of the primary outcome (i.e., AF inducibility), secondary outcomes (e.g., histology, fibrosis, cytokines, etc.) were not analyzed separately for recipient sex. EPS, electrophysiological study, EV, extracellular vesicle, veh, vehicle.

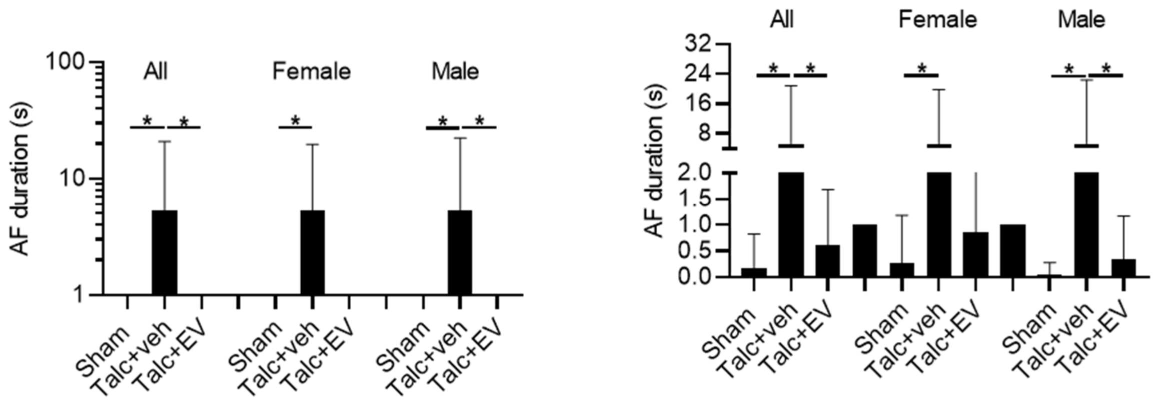

Supplemental Figure 4. Effect of EVs on AF duration with animals free of AF allocated a value of zero AF duration. Left panel reproduces Figure 3D in the manuscript. Right panel uses a standard y axis to highlight the values in sham or EV treated animals. *p<0.05. One-way Kruskal-Wallis ANOVA with individual-mean comparisons by Dunn's multiple comparisons test. AF, Atrial fibrillation; EV, extracellular vesicles; veh, vehicle.

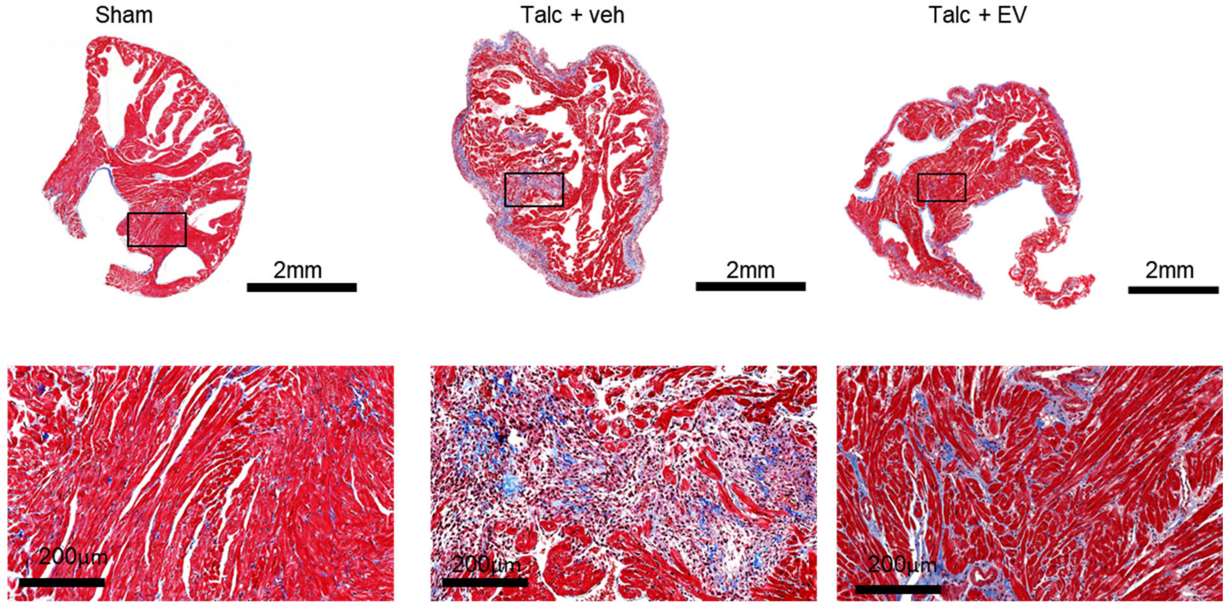

Supplemental Figure 5. Human atrial extracellular vesicle (EV) or vehicle (veh) effects on atrial fibrosis 3 days after surgery. Representative images of atrial tissue sections after Masson's Trichrome staining. Scale bar, 2 mm or 200 µm, as indicated.

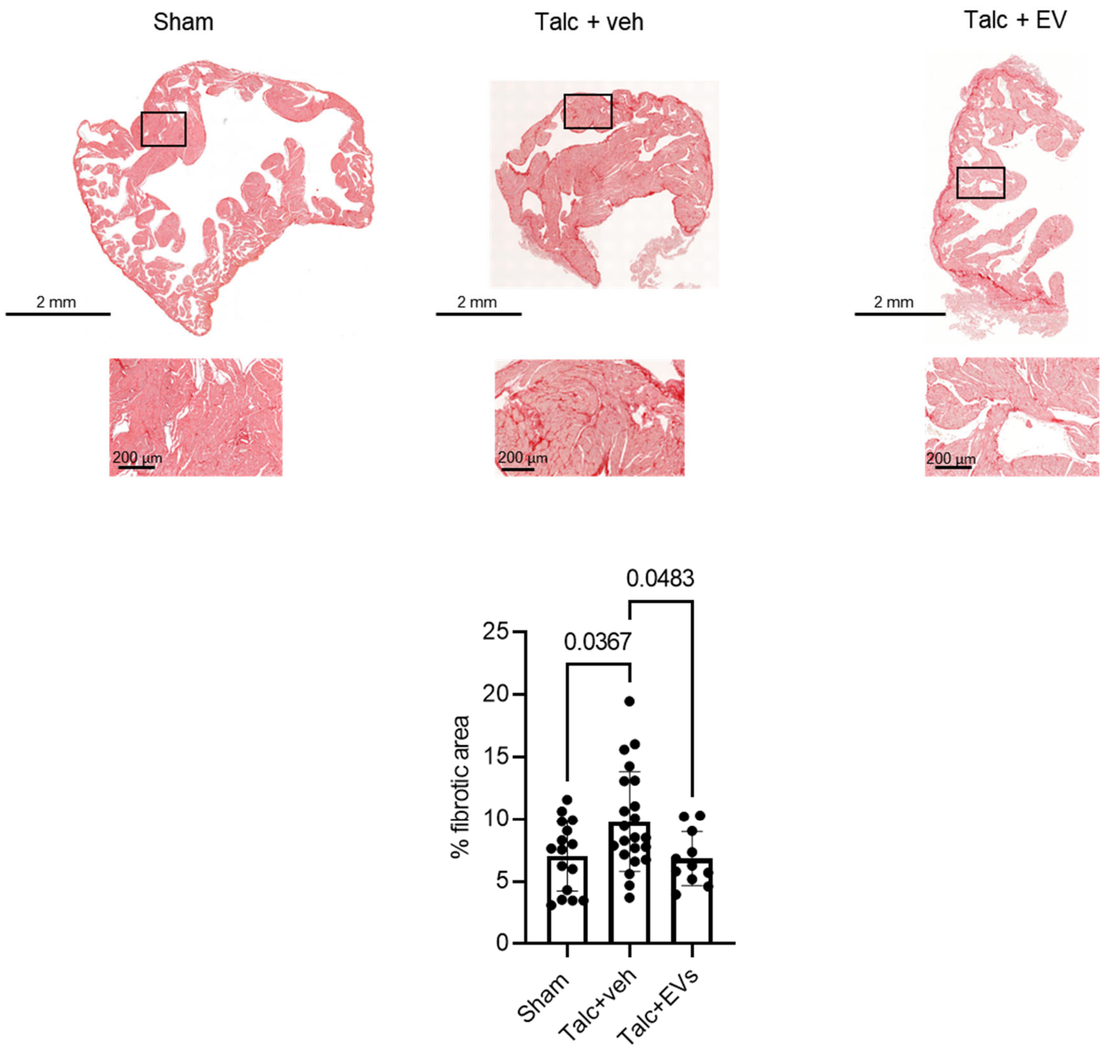

Supplemental Figure 6. Human atrial extracellular vesicle (EV) or vehicle (veh) effects on atrial fibrosis 3 days after surgery. Representative images of atrial tissue sections after picrosirius red staining. One-way ANOVA with individual-mean comparisons by Bonferroni's multiple two-tailed comparisons test. Scale bar, 2 mm or 200 µm, as indicated.

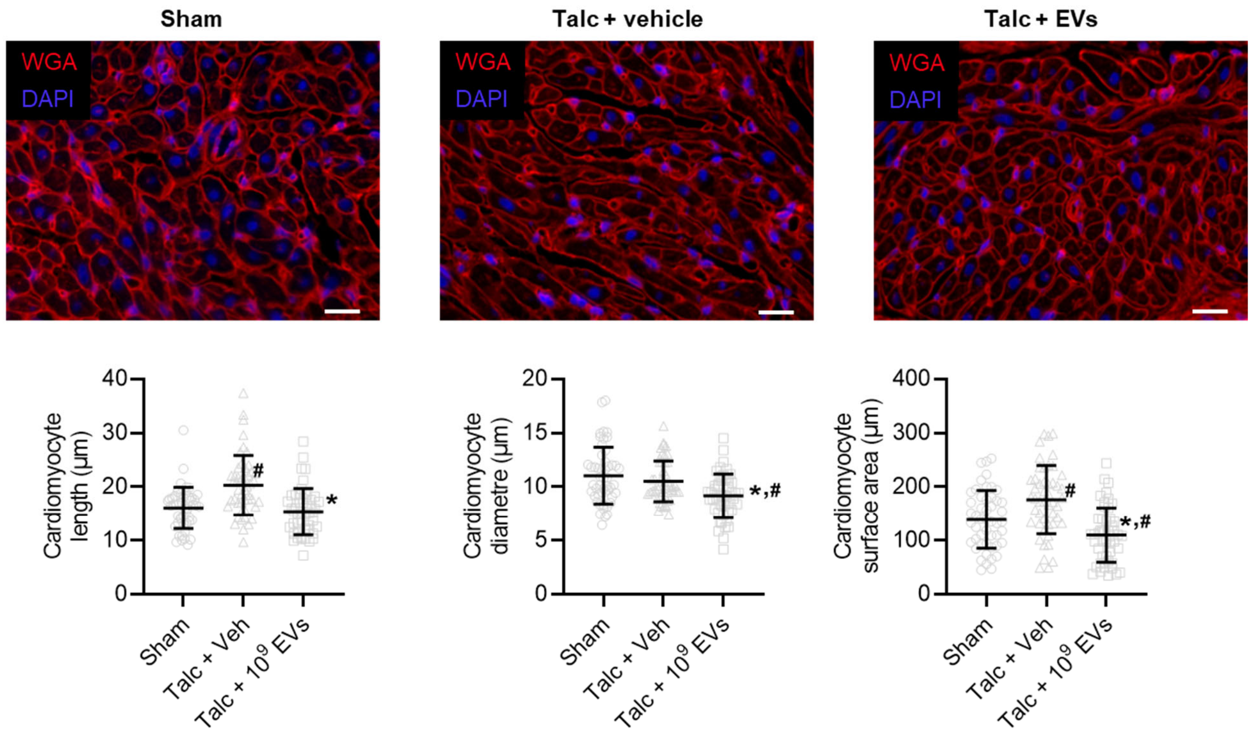

Supplemental Figure 7. Intramyocardial injection of human extracellular vesicles (EVs) reduce cardiomyocyte size in a model of rat sterile pericarditis. Effect of pericarditis and intramyocardial injection of EVs on cardiomyocyte size quantified using histological analysis plasma membrane border after staining with using wheat germ agglutinin (WGA) and 4′,6-diamidino-2-phenylindole (DAPI). Scale bar 20 µm. One-way ANOVA with individual-mean comparisons by Šídák's multiple comparisons test. *P<0.05 vs. talc + vehicle. #P<0.05 vs. sham. EV, extracellular vesicles; veh, vehicle.

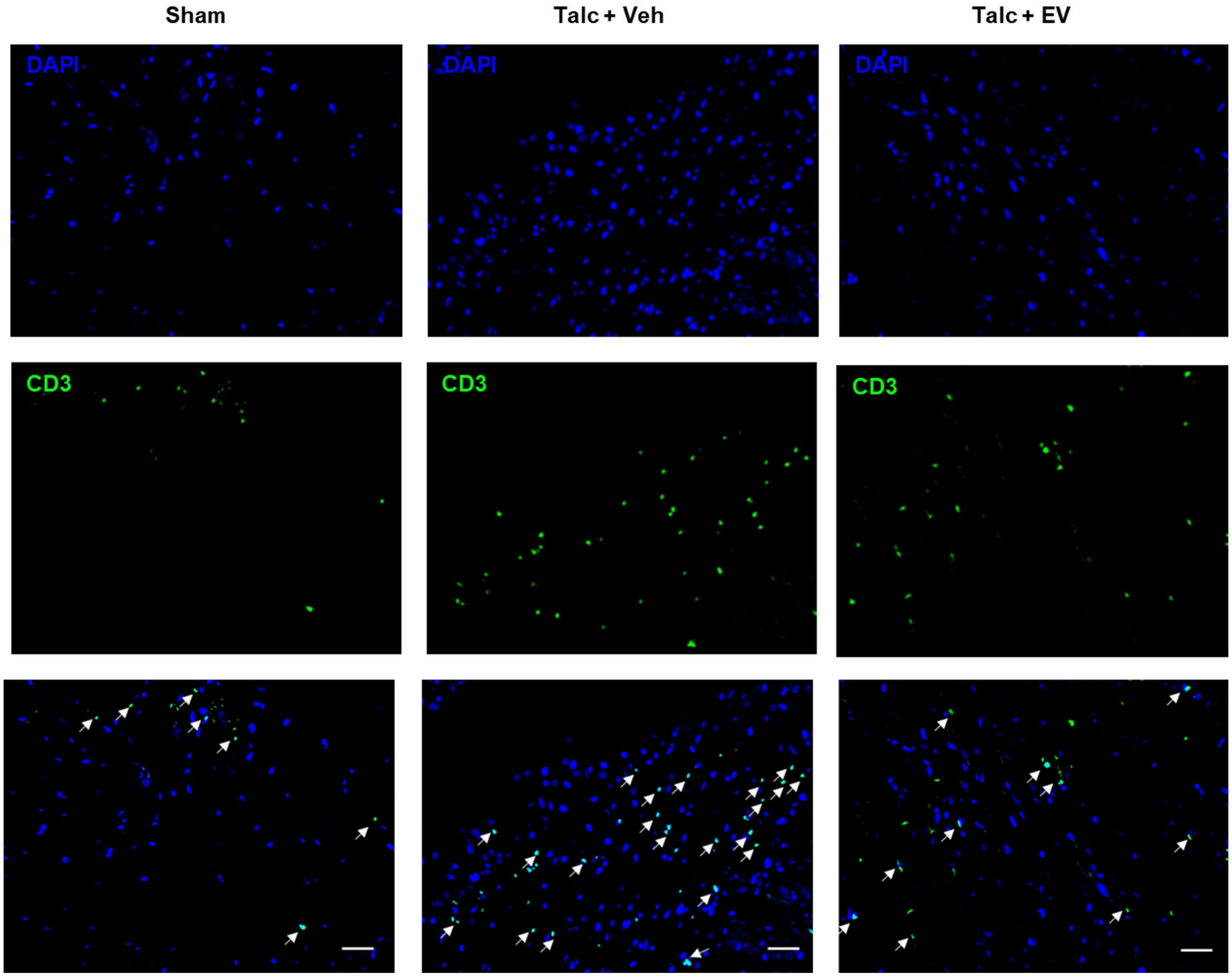

Supplemental Figure 8. Effect of explant-derived cell (EDC) extracellular vesicles (EVs) on cytotoxic T cells (CD3) within the treated atria. Representative images showing cytotoxic T cells (CD3) found within treated atria after sham procedure, application of talc and vehicle (veh) or after application of talc and EVs. Positive cells are denoted with a white arrow. Scale bar 100 μM.

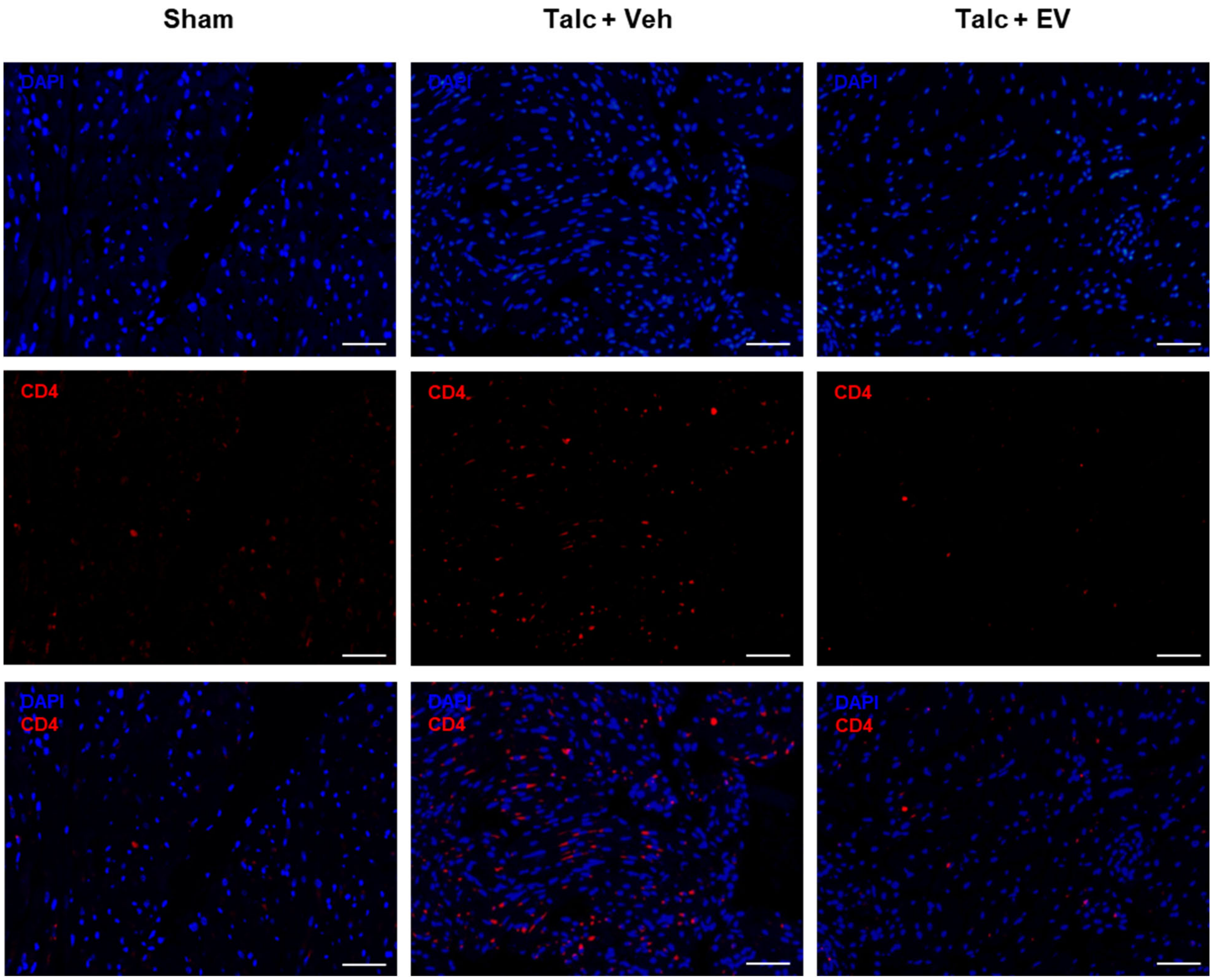

Supplemental Figure 9. Effect of explant-derived cell (EDC) extracellular vesicles (EVs) on T helper cells (CD4) within the treated atria. Representative images showing T helper cells (CD4) after sham procedure, application of talc and vehicle (veh) or after application of talc and EVs. Scale bar 50 μM.

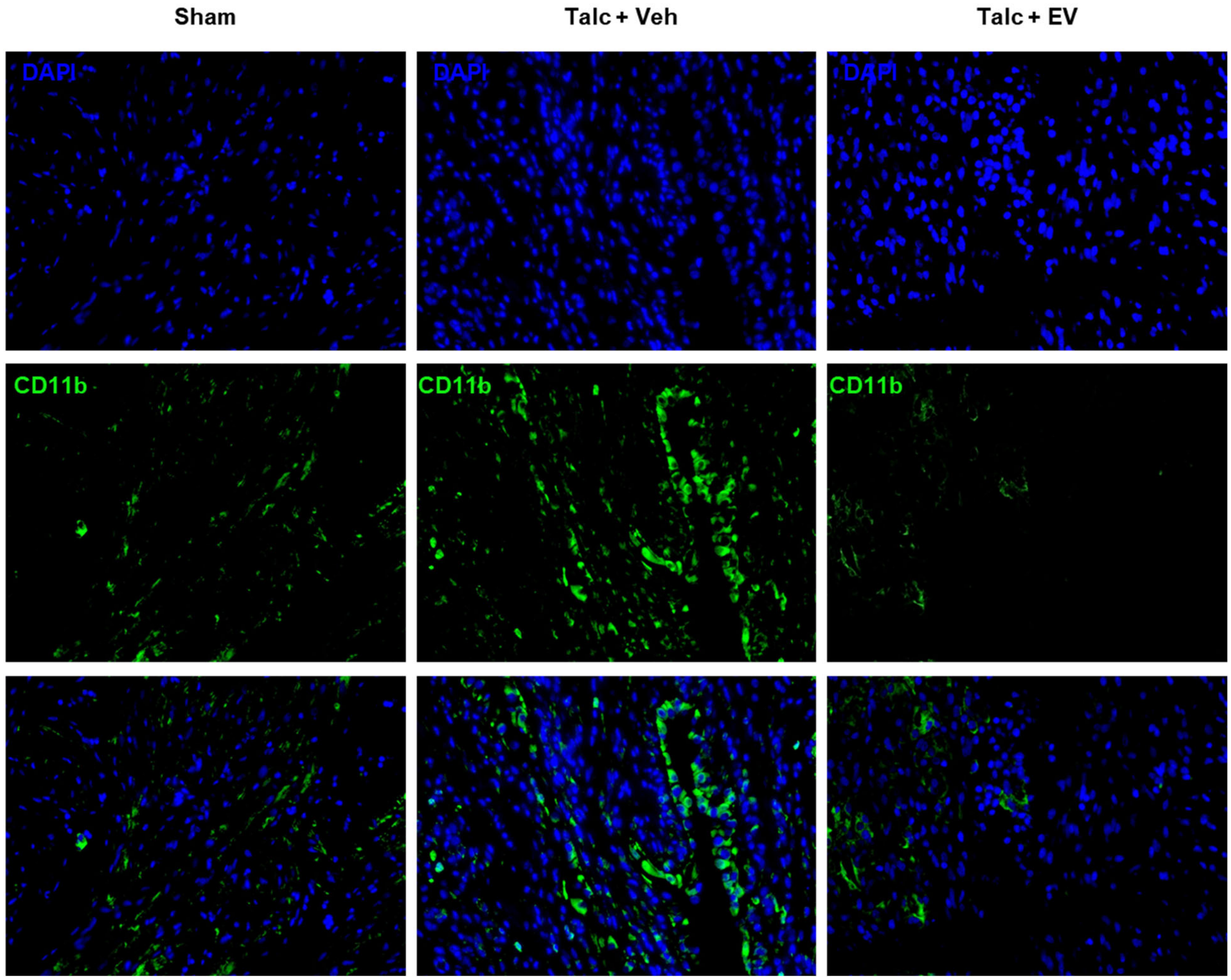

Supplemental Figure 10. Effect of explant-derived cell (EDC) extracellular vesicles (EVs) on neutrophils (CD11b) within the treated atria. Representative images showing neutrophils (CD11b) found within treated atria after sham procedure, application of talc and vehicle (veh) or after application of talc and EVs. Scale bar 100 μM.

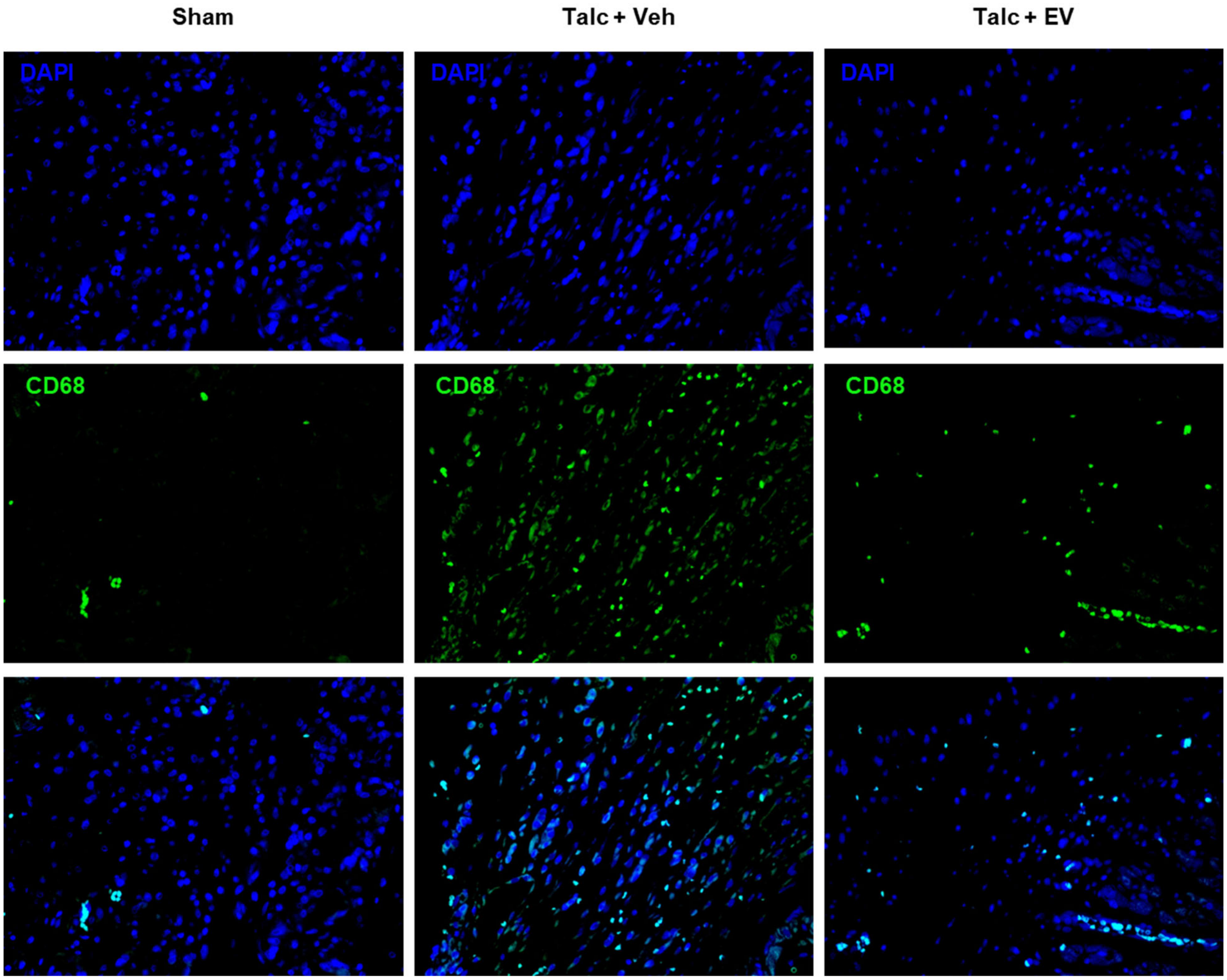

Supplemental Figure 11. Effect of explant-derived cell (EDC) extracellular vesicles (EVs) on pro-inflammatory M1 macrophages within the treated atria. Representative images showing pro-inflammatory M1 macrophages (CD68) found within treated atria after sham procedure, application of talc and vehicle (veh) or after application of talc and EVs. Scale bar 100 μM.

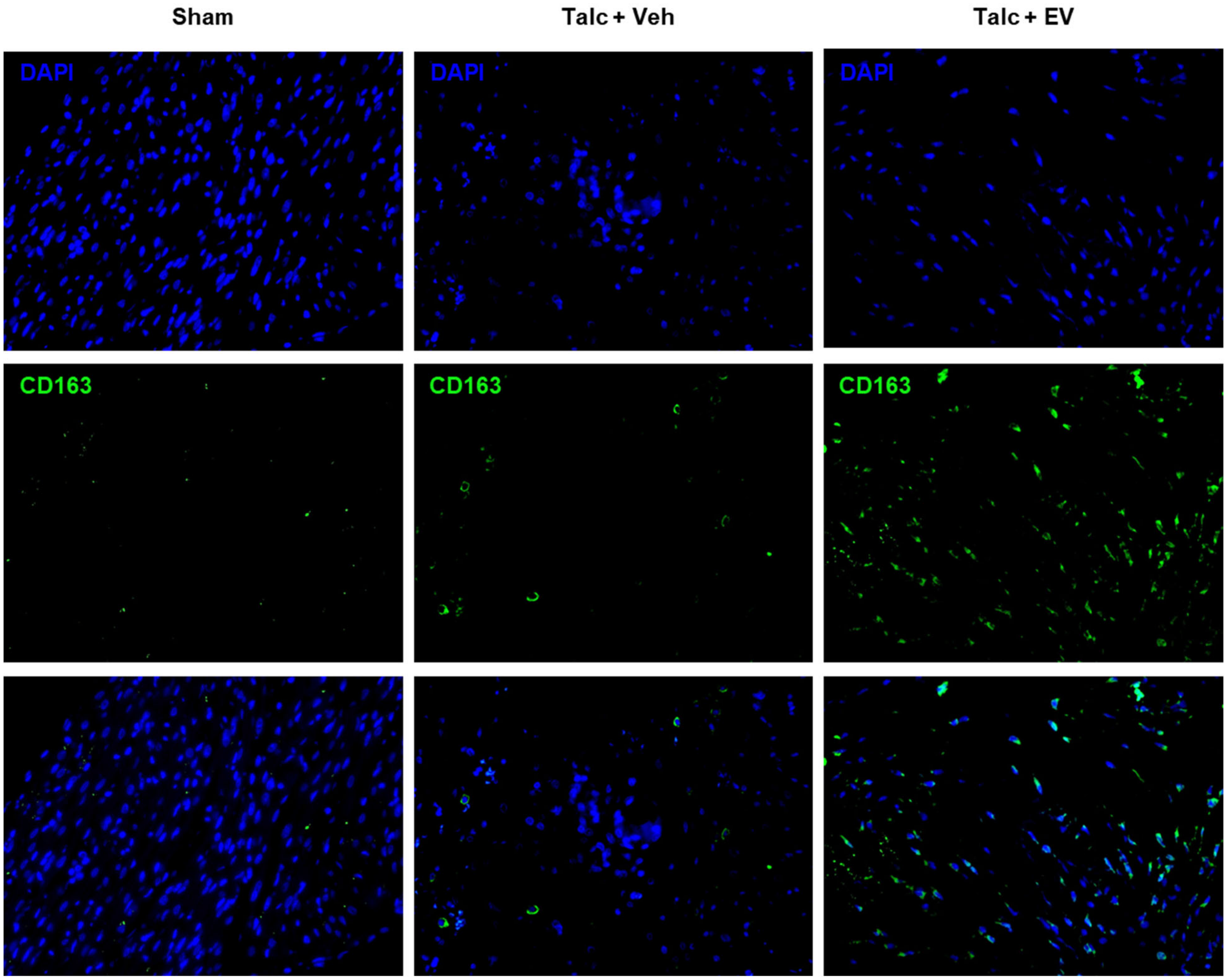

Supplemental Figure 12. Effect of explant-derived cell (EDC) extracellular vesicles (EVs) on anti-inflammatory M2 macrophages within the treated atria. Representative images showing anti-inflammatory M2 macrophages (CD163) found within treated atria after sham procedure, application of talc and vehicle (veh) or after application of talc and EVs. Scale bar 100 μM.

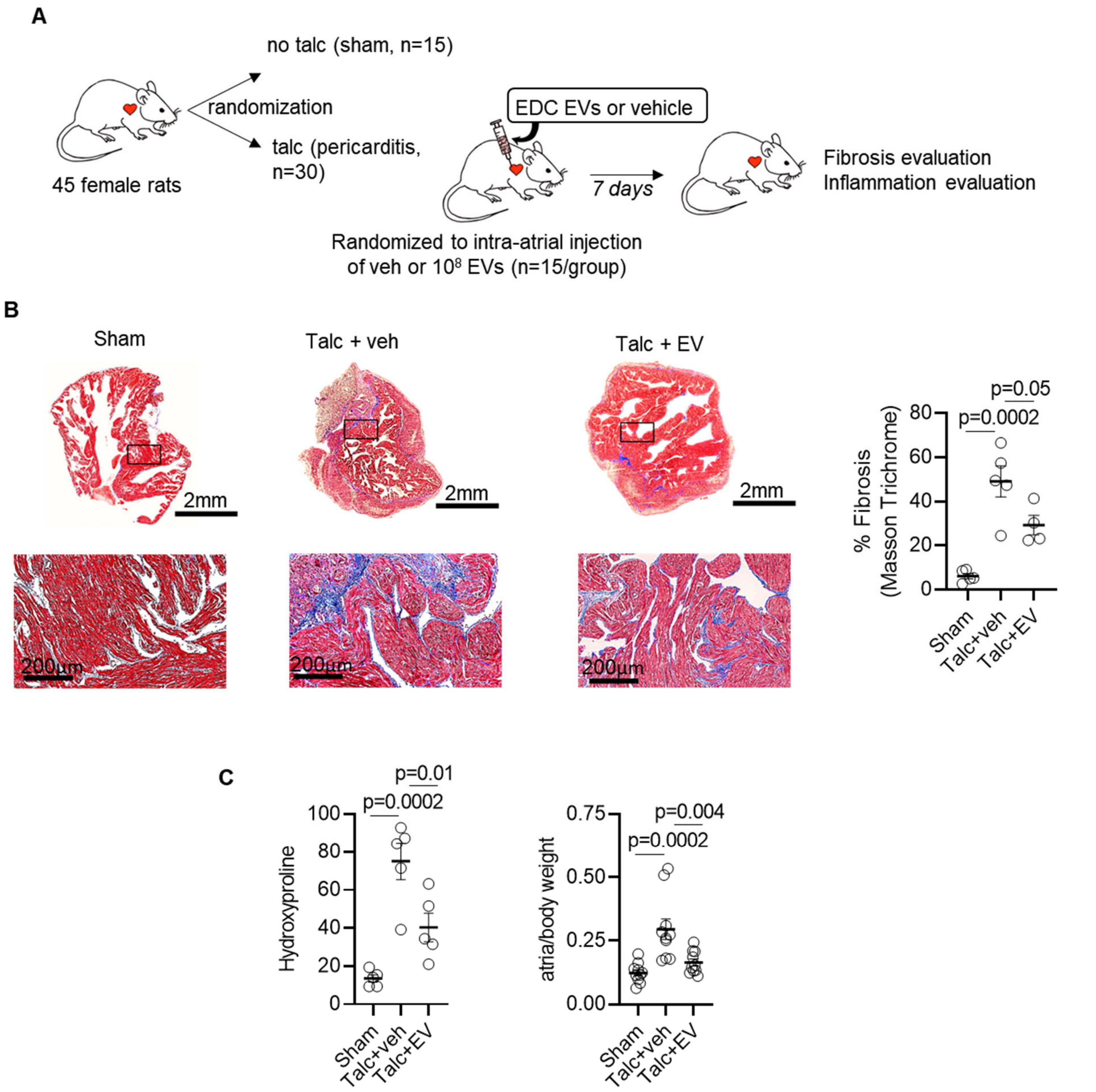

Supplemental Figure 13. Human atrial extracellular vesicle (EV) or vehicle (veh) effects on atrial structure and fibrosis 7 days after surgery. (A) Study schematic demonstrating the study design, numbers of animals included, group allocations and outcomes measured. **(B)** Representative images of atrial tissue sections after Masson's Trichrome staining. Quantitative analysis showing the effect of talc and EVs on inflammatory cell infiltration (n=5 biological replicates). **(C)** Effect of EV treatment on atrial fibrosis (n=5) and structure (n=5). One-way ANOVA with individual-mean comparisons by Bonferroni's multiple two-tailed comparisons test. Scale bar, 2 mm or 200 µm, as indicated.

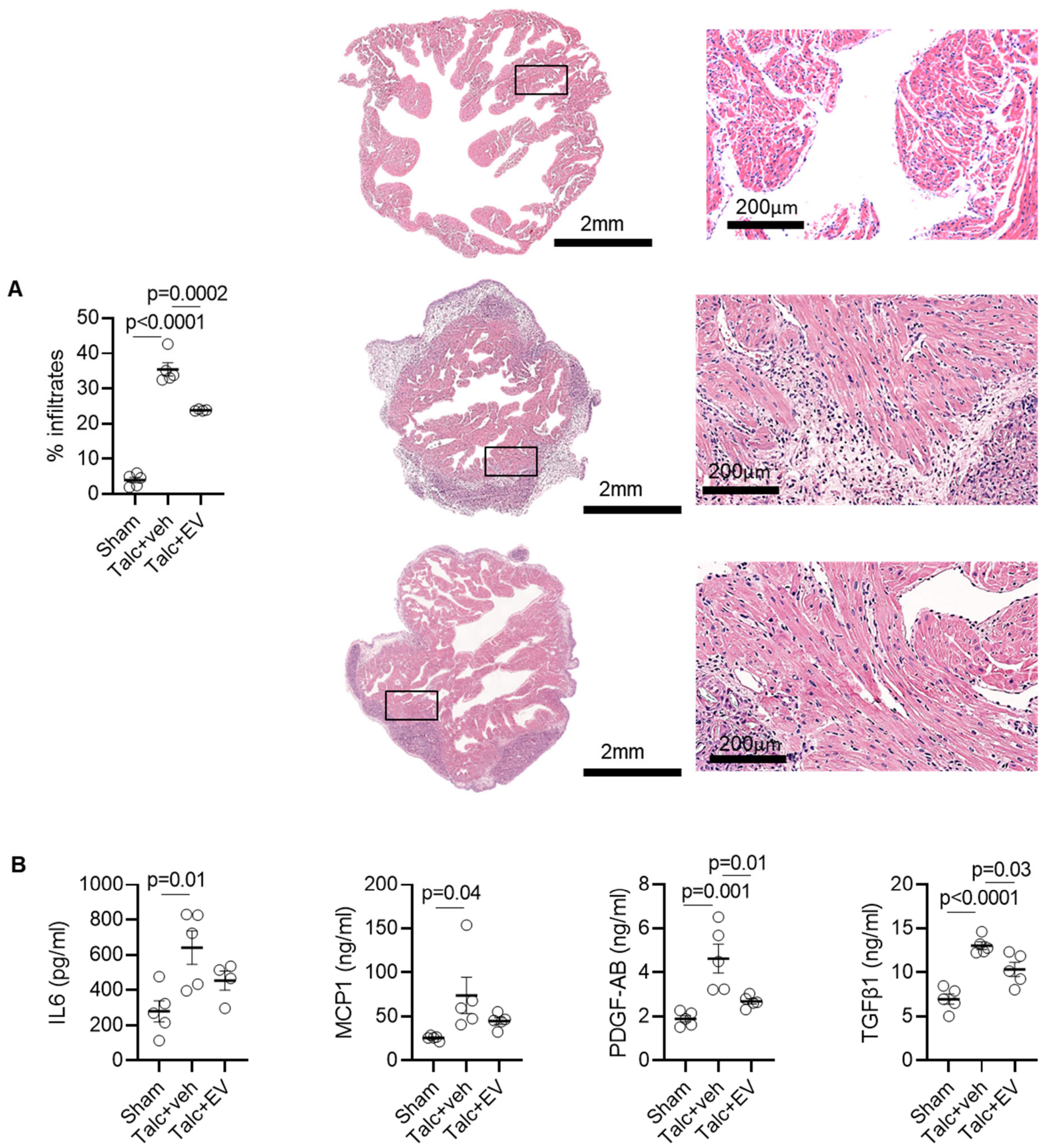

Supplemental Figure 14. Human atrial extracellular vesicle (EV) or vehicle (veh) effects on atrial inflammation 7 days after surgery. (**A**) Representative images of atrial tissue sections after hematoxylin and eosin staining showing the effect of talc and extracellular vesicles (EVs) on inflammatory cell infiltration. Quantitative analysis showing the effect of talc and EVs on inflammatory cell infiltration (n=5 biological replicates). (**B**) Effect of talc and EVs on interleukin 6 (IL6), monocyte chemoattractant protein 1 (MCP1), platelet-derived growth factor AB (PDGF-AB) and transforming growth factor beta 1 (TGFβ1) expression (n=5 biological replicates). One-way ANOVA with individual-mean comparisons by Bonferroni's multiple two-tailed comparisons test. Scale bar, 2 mm or 200 µm, as indicated.

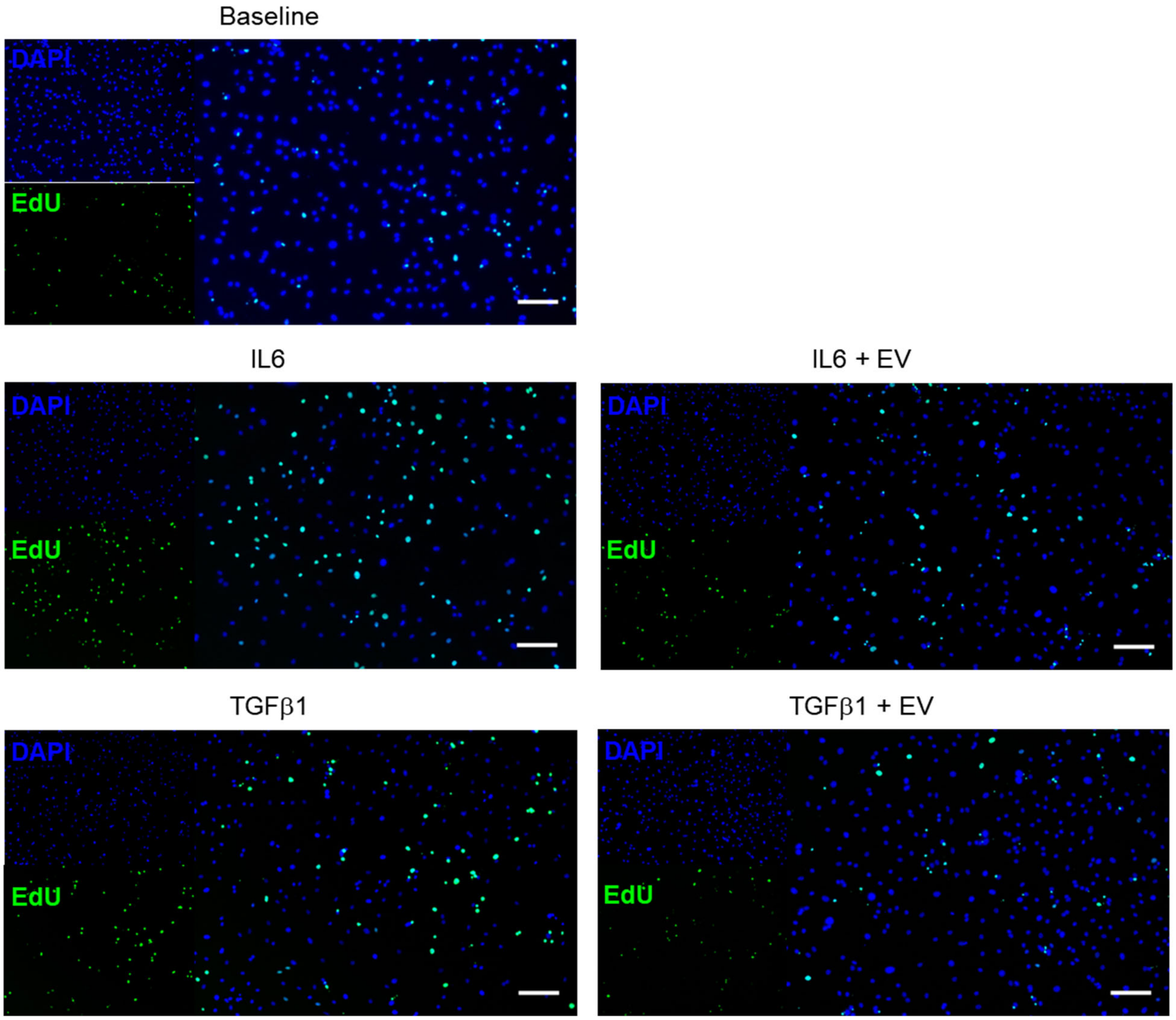

Supplemental Figure 15. Effect of explant-derived cell (EDC) extracellular vesicles (EVs) on proliferation. Representative images showing 5-ethynyl-2´-deoxyuridine (EDU) and 4′,6-diamidino-2-phenylindole (DAPI) atrial fibroblasts at baseline and after treatment with interleukin 6 (IL6), transforming growth factor beta 1 (TGFβ1) and/or EDC EVs (n=5 biological replicates). Scale bar 200 μM
